# Unraveling the Photoactivation Mechanism of a Light-Activated Adenylyl Cyclase Using Ultrafast Spectroscopy Coupled with Unnatural Amino Acid Mutagenesis

**DOI:** 10.1021/acschembio.2c00575

**Published:** 2022-08-29

**Authors:** Jinnette Tolentino Collado, James N. Iuliano, Katalin Pirisi, Samruddhi Jewlikar, Katrin Adamczyk, Gregory M. Greetham, Michael Towrie, Jeremy R. H. Tame, Stephen R. Meech, Peter J. Tonge, Andras Lukacs

**Affiliations:** † Department of Chemistry, Stony Brook University, New York, New York 11794, United States; ‡ Department of Biophysics, Medical School, University of Pecs, Szigeti Street 12, Pecs 7624, Hungary; § School of Chemistry, University of East Anglia, Norwich NR4 7TJ, U.K.; ∥ Central Laser Facility, Research Complex at Harwell, Rutherford Appleton Laboratory, Didcot OX11 0QX, U.K.; ⊥ Drug Design Laboratory, Graduate School of Medical Life Science, Yokohama City University, Tsurumi, Yokohama 230-0045, Japan

## Abstract

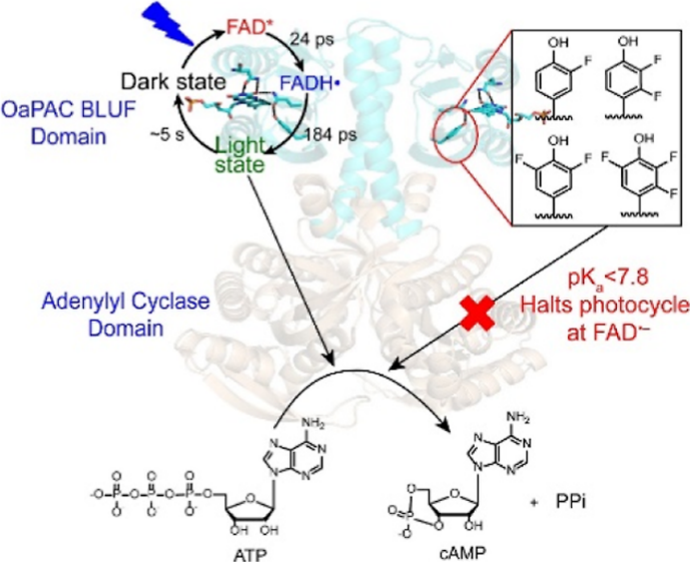

The hydrogen bonding network that surrounds the flavin in blue light using flavin adenine dinucleotide (BLUF) photoreceptors plays a crucial role in sensing and communicating the changes in the electronic structure of the flavin to the protein matrix upon light absorption. Using time-resolved infrared spectroscopy (TRIR) and unnatural amino acid incorporation, we investigated the photoactivation mechanism and the role of the conserved tyrosine (Y6) in the forward reaction of the photoactivated adenylyl cyclase from *Oscillatoria acuminata* (OaPAC). Our work elucidates the direct connection between BLUF photoactivation and the structural and functional implications on the partner protein for the first time. The TRIR results demonstrate the formation of the neutral flavin radical as an intermediate species on the photoactivation pathway which decays to form the signaling state. Using fluorotyrosine analogues to modulate the physical properties of Y6, the TRIR data reveal that a change in the p*K*_a_ and/or reduction potential of Y6 has a profound effect on the forward reaction, consistent with a mechanism involving proton transfer or proton-coupled electron transfer from Y6 to the electronically excited FAD. Decreasing the p*K*_a_ from 9.9 to <7.2 and/or increasing the reduction potential by 200 mV of Y6 prevents proton transfer to the flavin and halts the photocycle at FAD^•-^. The lack of protonation of the anionic flavin radical can be directly linked to photoactivation of the adenylyl cyclase (AC) domain. While the 3F-Y6 and 2,3-F_2_Y6 variants undergo the complete photocycle and catalyze the conversion of ATP into cAMP, enzyme activity is abolished in the 3,5-F_2_Y6 and 2,3,5-F_3_Y6 variants where the photocycle is halted at FAD^•-^. Our results thus show that proton transfer plays an essential role in initiating the structural reorganization of the AC domain that results in AC activity.

## Introduction

**Figure 1:**
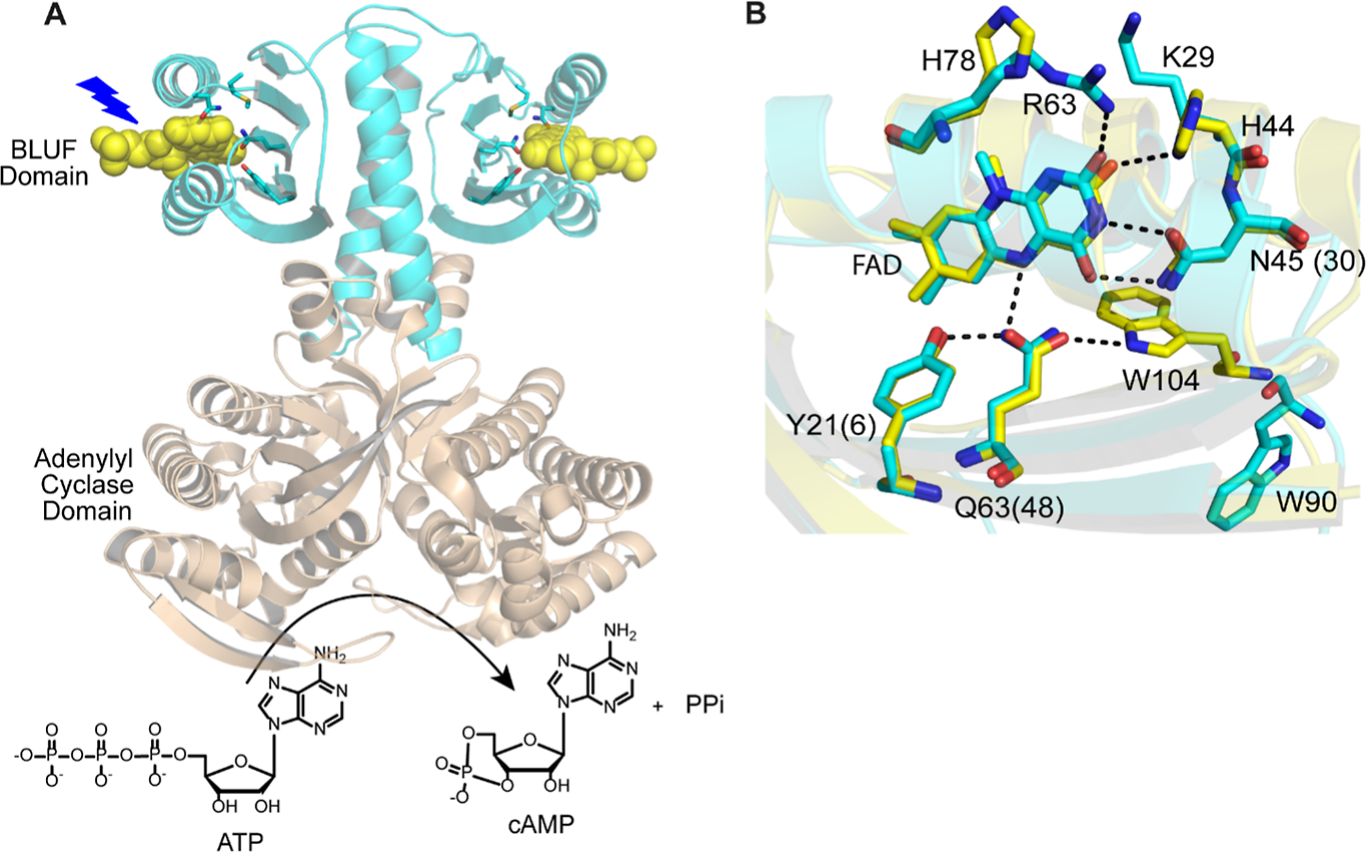
Structure of OaPAC and the flavin binding pocket. (A) OaPAC is a homodimer composed of a BLUF domain (cyan color) linked to an AC domain (sand color) responsible for converting ATP into cAMP and PPi in a light-dependent manner (PDB: 5x4t).^18^ (B) Hydrogen-bonding network that surrounds the isoalloxazine ring of the FAD. The structure of OaPAC (cyan) has been superimposed on the structure of AppA (yellow; PDB: 1YRX) where the tryptophan (W104) is in the Trp_in_ conformation.^12^ Residue numbers are for AppA, while those in parentheses are for OaPAC.

Adenylyl cyclases (ACs) are an important class of enzymes that catalyze the formation of cyclic adenosine 3′,5′-monophosphate (cAMP) from ATP (Figure 1). cAMP is a second messenger that plays a crucial signaling role in many organisms, and the photoactivated adenylate cyclases (PACs) represent a subgroup of AC enzymes where the G-protein independent synthesis of cAMP is controlled by light. Early work led to the discovery of PACs in the unicellular flagellate *Euglena gracilis* (EuPAC),^1,2^ and the sulfide-oxidizing bacterium *Beggiatoa* sp. (bPAC),^3,4^ where AC activity is controlled by a blue light using the flavin adenine dinucleotide (BLUF) domain. bPAC was found to have a low activity in the dark and a 100-fold increase in activity upon blue light irradiation.

PACs are attractive optogenetic tools given that the production of cAMP can be controlled by light. For example, EuPAC was expressed in the neurons of the marine gastropod *Aplysia* enabling photocontrol of neuron stimulation,^5^ while bPAC was used in transgenic mice to restore and control the flagellar beat of sperm by light.^6^ Another promising PAC (OaPAC) was discovered recently in the photosynthetic cyanobacterium *Oscillatoria acuminata* which shows the lowest activity in the dark among PAC proteins, enabling finer control of photoinduced cAMP production.^7^ OaPAC, Figure 1A, is a 366-aa residue homodimer comprising an N-terminal BLUF domain and C-terminal AC domain and shows ∼57% sequence homology to bPAC. The full-length structures in both dark and light states were recently reported.^7^

BLUF domain proteins are found in many organisms and are involved in light-dependent processes such as phototaxis, photophobia, and photosynthesis.^8,9^ AppA was the first BLUF protein to be discovered and is involved in the light and redox regulation of photosynthetic gene expression in *Rhodobacter sphaeroides*.^10^ Under low light conditions and low oxygen levels, AppA binds to the transcriptional anti-repressor PpsR. Upon blue light illumination, photoactivation results in a conformational change in AppA that leads to the dissociation of the AppA–PpsR complex enabling PpsR to repress the transcription of genes encoding photosynthetic proteins. The initial event in BLUF photoactivation involves the reorganization of a hydrogen bonding network that surrounds the flavin chromophore resulting in a ∼10 nm red shift of the *S*_0_ → *S*_1_ flavin transition at ∼450 nm. Site-directed mutagenesis has demonstrated that a conserved tyrosine and glutamine (Y21 and Q63 in AppA) are essential for photoactivation, and there is a general agreement that the red shift in the electronic transition is caused by the formation of a hydrogen bond to the flavin C4=O from the glutamine in the light-adapted state. Early structural studies supported a model which involves a 180° rotation of glutamine,^11,12^ and based on experiments on the Slr1694 BLUF domain (PixD), it has been proposed that rotation of the glutamine is triggered by electron transfer from the conserved tyrosine to the flavin.^13^ In addition, we have also suggested that the hydrogen bond rearrangement is driven by keto-enol tautomerism of the glutamine,^14,15^ a model that is supported by isotope-edited Fourier transform infrared (FTIR) spectroscopy and theoretical calculations.^16,17^

The X-ray structure of OaPAC (PDB: 5x4t) reveals that the flavin binding pocket contains the same conserved residues found in other BLUF proteins.^18^ The isoalloxazine ring is surrounded by a conserved tyrosine (Y6), glutamine (Q48), and asparagine (N30), while a conserved methionine (M92) is also present in the flavin binding pocket (Figure 1B). The semi-conserved tryptophan (W90) is in the Trp_out_ conformation as also noted in the X-ray structure of the BLUF photoreceptor PixD (PDB: 2HFN)^19^ and the recently solved X-ray structure of BlsA (PDB: 6W6Z).^20^ Comparison of the high-resolution crystal structures of wild-type OaPAC in its light and dark state reveals that upon light absorption, the Cγ–Cδ bond of glutamine (Q48) rotates about 40°, while the Nε2 atom of the Q48 moves away from N5 of the flavin toward the O4 of the flavin C4=O group enabling a second hydrogen bond to be formed C4=O. Ohki et al.^18^ proposed a possible mechanism involving tautomerization of the Q48 side chain and requiring the formation of two radicals, FADH^•^ and Y6^•^, that would decay within nanoseconds to the signaling state.

In this work, we employed fs–ms time-resolved infrared spectroscopy (TRIR) in combination with site directed and unnatural amino acid (UAA) mutagenesis to probe the photoactivation mechanism of OaPAC, a BLUF protein with a covalently attached output domain. Our studies illustrate that the excitation of FAD in wild-type OaPAC leads to a concerted proton coupled electron transfer (PCET) and the formation of the neutral flavin radical (FADH^•^) which reacts with Y^•^ radical to form the final light state where the flavin is in the oxidized form. Moreover, we interrogated the role of Y6 in PCET in the activation of wild-type OaPAC by incorporating fluorotyrosine analogues. In the TRIR experiments, only 3-FY6 OaPAC was able to form a light state but with slower kinetics compared to wild-type OaPAC, whereas excitation of 3,5-F_2_Y6, 2,3,5-F_3_Y6, and 2,3-F_2_Y6 OaPAC variants did not generate a detectable light state. In addition, we tested the activity of AC domain for the wild-type OaPAC and *n*-FY6 variants. Whereas 3-FY6 and 2,3-F_2_Y6 OaPAC have similar adenylate cyclase activity compared to the wild-type, the 3,5-F_2_Y6 and 2,3,5-F_3_Y6 OaPAC variants were inactive.

## Results

### UV–Visible Absorption and FTIR Difference Spectra of Wild-Type OaPAC

**Figure 2:**
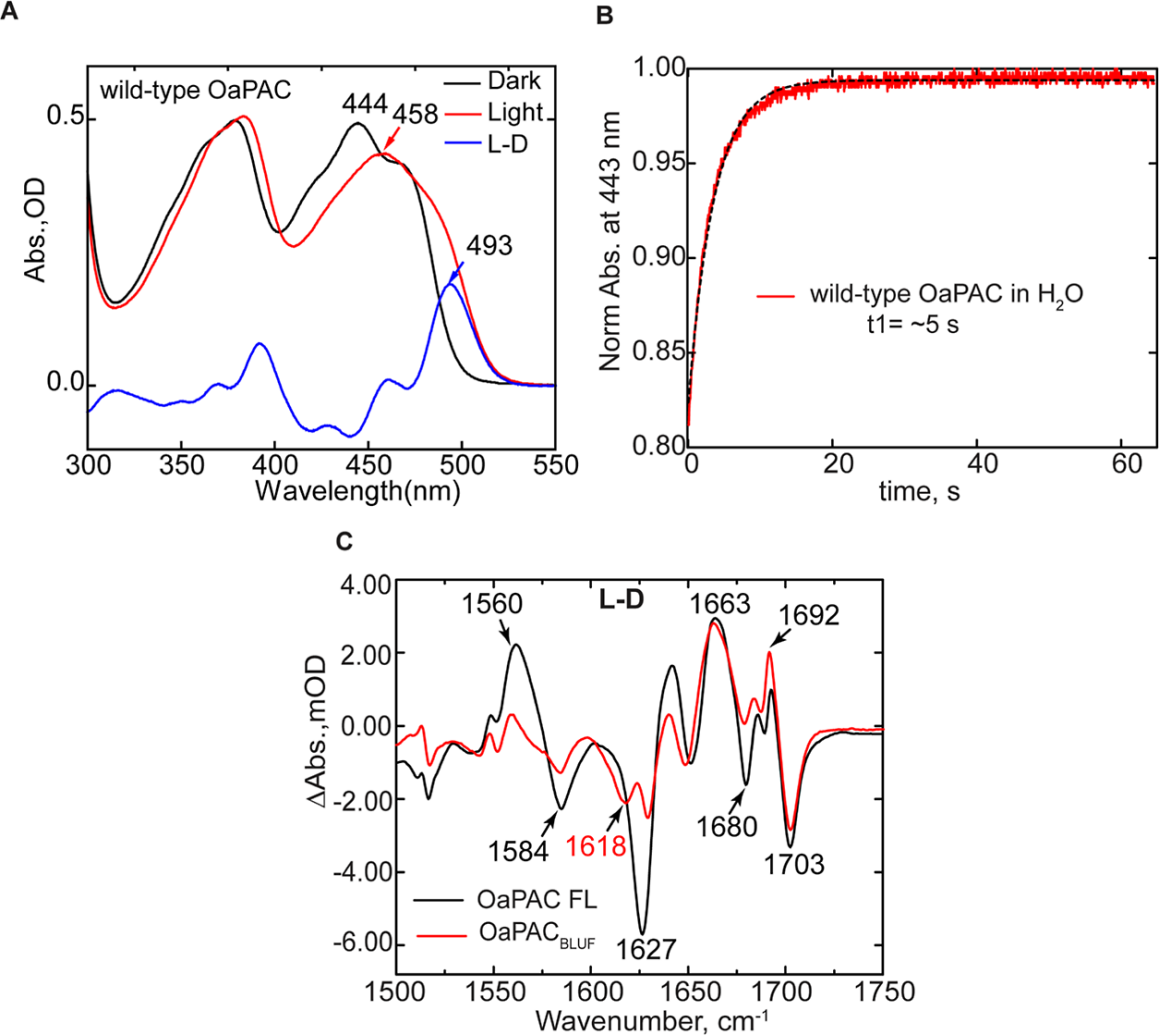
UV–visible absorption and FTIR difference spectra of wild-type OaPAC. (A) Steady-state absorption spectra for wild-type OaPAC in the dark and light. (B) Dark-state recovery of the band at 444 nm which has a time constant of ∼5 s. C. Comparison of the steady-state IR difference spectrum of full-length OaPAC (OaPAC FL) with OaPAC_BLUF_. The FTIR difference spectrum was obtained using 1 mM protein in a 50 μm CaF_2_ cell. The L – D spectrum was generated by subtracting the IR spectrum of OaPAC acquired before irradiation from the spectrum acquired, while the sample was irradiated using a 455 nm mounted LED for at least 20 s.

Figure 2A shows the steady-state absorption spectrum of wild-type OaPAC obtained before and after irradiation with 450 nm blue light LED [∼500 μW of 455 (±10) nm light, 20 s illumination]. The wild-type protein exhibits a 14 nm red shift in the flavin absorption spectrum upon blue light irradiation, which is a characteristic of all photoactive BLUF proteins and is due to changes in hydrogen bonding around the flavin. The absorption difference spectrum (Figure 2A, blue spectrum) shows an absorbance maximum at 493 nm, associated with light-state formation. Once illumination is discontinued, the absorption spectrum relaxes back to that observed for the dark-state with a time constant of ∼5 s (Figure 2B). The rate of dark-state recovery for OaPAC is faster than that reported for other BLUF proteins including PixD (45 s), AppA_BLUF_ (23 min), and BlsA (8 min).^20,21^

The structural changes that accompany light-state formation were first studied using steady-state FTIR difference spectroscopy. The difference spectrum of OaPAC was generated by subtracting the dark-state FTIR spectrum from that obtained while the sample was continuously illuminated with 460 (±5) nm LED (Prizmatix, Ltd.) placed in the sample compartment and focused onto the cell using an objective. The light minus dark (L – D) IR difference spectrum from 1800–1100 cm^–1^ is shown in Figure 2C, where positive and negative bands are attributed to the light-induced and ground-state vibrational modes, respectively. At higher wavenumbers, the L – D difference spectrum of wild-type OaPAC is similar to that observed for other BLUF proteins where two prominent bands at 1692(+)/1703(−) cm^–1^ are assigned to a shift in the C4=O carbonyl vibration of the flavin, consistent with an increase in hydrogen bonding in the light state.^14,22^ The lower wavenumber modes in the 1550–1650 cm^–1^ region are assigned to changes in the vibrations of the protein backbone, in particular the β5 strand which is important for signal transduction.^3,23,24^ The difference spectrum of the OaPAC BLUF domain lacking the AC domain (1–141 aa; OaPAC_BLUF_) was also acquired and exhibits significantly lower intensity in the difference bands between 1550 and 1650 cm^–1^ region, especially at 1627 cm^–1^ (Figure 2C). Thus, compared to the full-length protein, the light-induced structural changes of the protein backbone in the OaPAC_BLUF_ are suppressed. A similar observation was reported in the comparison of light-induced structural changes in the full-length protein and BLUF domain of the cyclic-di-GMP phosphodiesterase YcgF/Blrp.^24^ In addition, a similar study was also performed on the full-length and isolated BLUF domain of bPAC. Interestingly, a large positive signal was observed in the frequency range expected for α-helical amide I vibrations around 1650 cm^–1^ in the full-length bPAC, suggesting that the photoactivation of the cyclase domain involves the formation of helical structures. In the FTIR difference spectrum of wild-type OaPAC, we do not observe a positive peak in the 1650 cm^–1^ region, indicating that light-induced structural changes of wild-type OaPAC might differ from bPAC in the signal transduction mechanism. This is not unexpected as the C-terminal region of bPAC is shorter compared to OaPAC; interestingly, the shorter C-terminal region in bPAC results in a more efficient cAMP production compared to OaPAC.^25^

### TRIR and Time-Resolved Multiple Probe Spectroscopy of Wild-Type OaPAC

**Figure 3:**
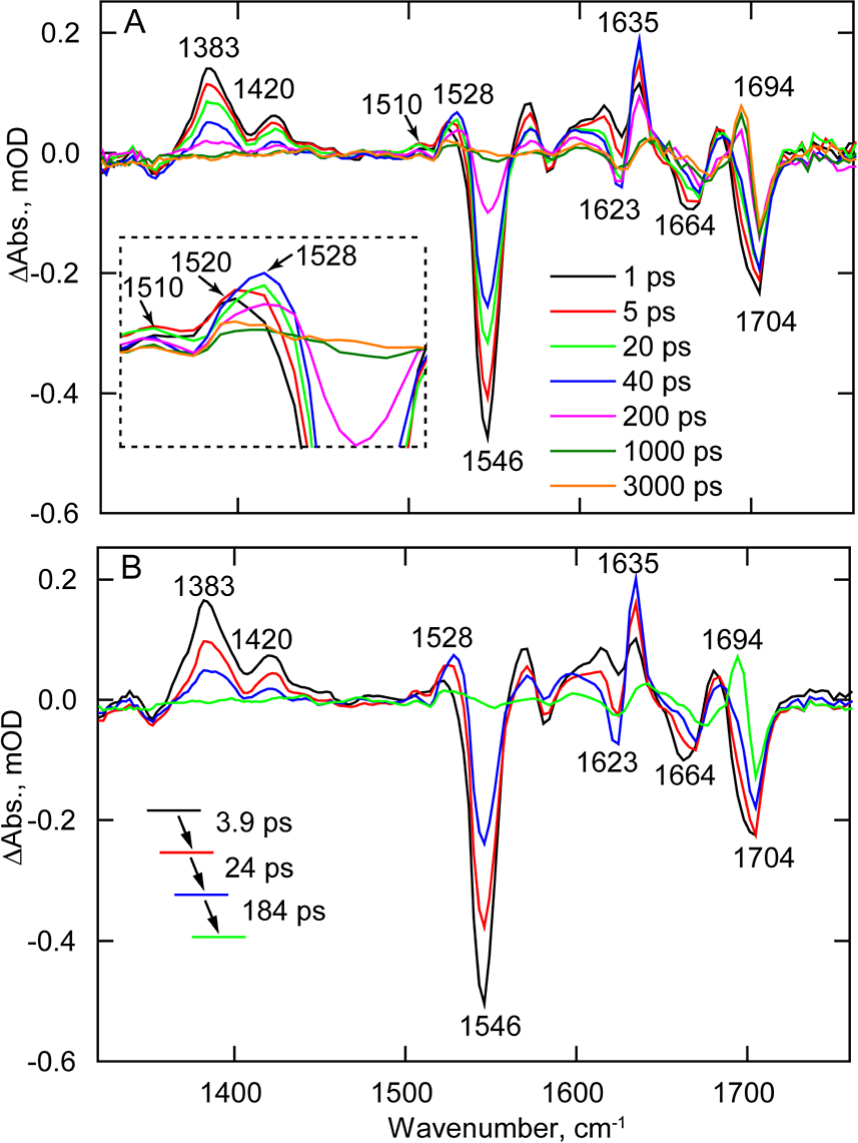
TRIR Spectra of the wild-type OaPAC dark state. (A) Temporal evolution of wild-type OaPAC spectra recorded between 100 fs and 3 ns after 450 nm excitation. (B) EADS of wild-type OaPAC obtained from a global fit of the TRIR data in A. Transients assigned to FADH^•^ (1528 cm^–1^), FAD^•–^ (1520 cm^–1^), and Tyr^•^ (1510 cm^–1^) are shown in the inset.

Figure 3 shows the temporal evolution of the TRIR spectra of the dark-adapted wild-type OaPAC upon excitation of the isoalloxazine chromophore by a 450 nm sub-100 fs pump pulse. The TRIR difference spectra comprised negative and positive bands, where the negative bands (bleaches) are associated with depletion of the isoalloxazine singlet ground state, or with changes in the vibrational spectrum of the surrounding protein occurring either instantaneously as a result of chromophore excitation or due to subsequent structural dynamics.^26^ The positive bands (transient absorptions) arise due to vibrations of the electronically excited states of isoalloxazine, or to amino acid modes perturbed by electronic excitation, or to vibrational modes of products formed following excitation.

TRIR spectra of wild-type OaPAC (Figure 3A,B) are broadly similar to spectra of the other BLUF domain proteins we and others have previously studied.^23,27−29^Figure 3B shows the evolution-associated difference spectra (EADS) which represent the spectral evolution over time. To generate the EADS, TRIR data (Figure 3A) were globally analyzed in Glotaran using a sequential decay model of four components in which three of the lifetime components were allowed to vary, while the fourth component was kept constant (indicating a product with effectively infinite lifetime) yielding three time constants of 3.9, 24, and 184 ps (Table 1). The first EADS (Figure 3B, black line) shows the instantaneously formed excited state of FAD (FAD*) in wild-type OaPAC. The excited state is characterized by transient bands at 1383 and 1420 cm^–1^, and ground state bleaches at ∼1546, ∼1664, and ∼1704 cm^–1^, which are assigned to the C10a–N1, C2=O, and C4=O flavin modes, respectively.^28,30^ The first EADS also shows a band at 1520 cm^–1^, indicating that the FAD^•^ intermediate is formed at very early times post excitation (Figure 3A, inset).^31,32^ The second EADS (Figure 3B, red spectrum) forms with a 3.9 ps time constant and shows that FAD* is decaying, while the ground state is already recovering. Significantly, this spectrum is characterized by the appearance of a new transient at ∼1528 cm^–1^ which is a vibrational marker for the neutral semiquinone FADH^•^ species (NSQ) that is also observed in flavodoxin and glucose oxidase.^32,33^ In EADS3, the FADH^•^ transient at 1528 cm^–1^ increases in amplitude with a time constant of 24 ps and relaxes in 184 ps. On this timescale the FAD* continues to decay, consistent with the complex multiexponential kinetics observed in other BLUF domains,^29^ and there is a similarly complex evolution in the region of the FAD C=O bleach modes, suggesting underlying dynamics in protein modes. Simultaneously with FADH^•^ formation and decay, we observe the formation of another transient at 1635 cm^–1^ coinciding with an increase of a bleach at 1623 cm^–1^. The 1623 (−)/1635 (+) cm^–1^ modes can correspond to another flavin radical mode and/or a tyrosine radical.^32^ The fourth EADS (Figure 3B, green line) represents the final non-decaying component which is formed around 184 ps. The main feature in EADS4 is the formation of the transient at ∼1694 cm^–1^, which reflects the formation of the light state; this vibrational mode is assigned to the C4=O vibration of the flavin. The downshift from 1704 to 1694 cm^–1^ reflects the reorganization of the hydrogen bonds around the flavin after light excitation and the decrease in frequency is consistent with the formation of a second hydrogen bond to C4=O.

**Table 1:** Time Constants from TRIR Data for Wild-Type OaPAC and *n*-FY6 OaPAC Variants

	τ_1_/ps	τ_2_/ps	τ_3_/ps
wild-type OaPAC^a^	3.9	24	184
3-FY6 OaPAC^a^	3.1	32	510
2,3-F_2_Y6 OaPAC^b^	52	2079	infinite
3,5-F_2_Y6 OaPAC^b^	21.5	1354	infinite
2,3,5-F_3_Y6 OaPAC^b^	117	1682	infinite

a The TRIR data of wild-type OaPAC and 3-FY6 OaPAC were globally analyzed in Glotaran using three time constants to adequately describe the data.

b For 2,3-F_2_Y6, 3,5-F_2_Y6, and 2,3,5-F_3_Y6 OaPAC, only two time constants were used to fit the data followed by an “infinite” lifetime extending beyond the time scale of the TRIR measurements.

For wild-type OaPAC, the time scale of TRIR is not sufficient to observe the full evolution of the protein modes leading to the final light state, thus we also performed time-resolved multiple probe spectroscopy (TRMPS) to further monitor the protein dynamics (Figure S1). Superimposition of the 3 ns TRIR spectrum with the 100 μs TRMP spectrum and the steady-state FTIR difference spectrum (Figure S1) reveals that while the important changes in the steps of photocycle dynamics have occurred within the first 3 ns, further evolution occurs beyond 100 μs which exceeds the time window of TRMPS instrumentation and is likely due to secondary structural changes in the AC domain, most notably in the 1550–1660 cm^–1^ region. Tokonami et al. performed transient absorption (TA) measurements on the full-length OaPAC and observed a slow reaction phase in the tens of milliseconds reflecting a conformational change at the C-terminus domain, which is consistent with this result.^34^

### Time-Dependent Evolution of TRIR Bands

**Figure 4:**
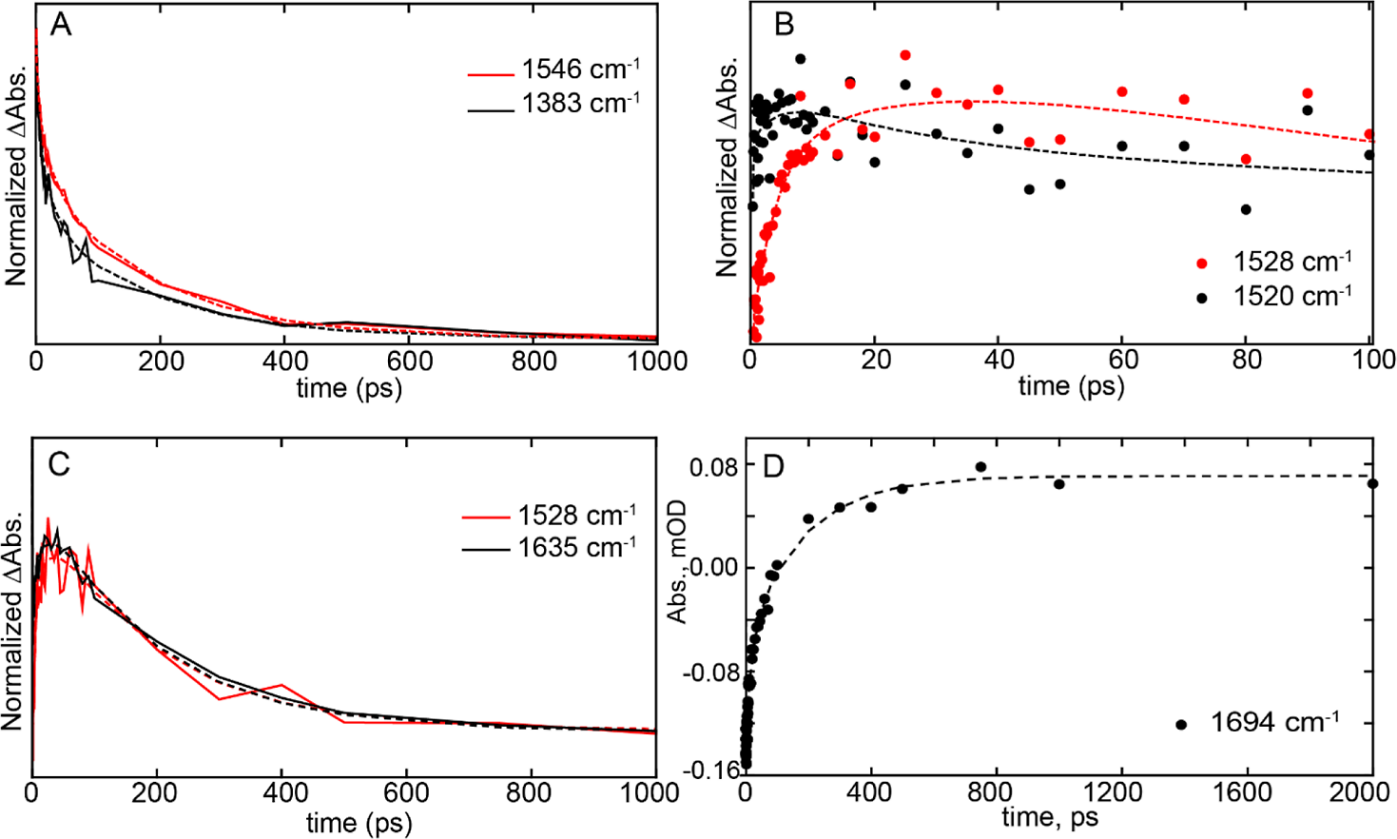
Time-dependent evolution of TRIR bands. (A) Kinetic traces of the excited state decay (1384 cm^–1^) and the ground-state recovery (1546 cm^–1^, shown inverted for comparison). (B) Comparison of the rise and decay of radical intermediates in wild-type OaPAC. (C) Comparison of the rise and decay of radical transient at 1528 cm^–1^ with transient evolving at 1635 cm^–1^. (D) Kinetic trace of the 1694 cm^–1^ transient, assigned to light-state formation. Raw data are shown as solid lines or dots, while global fitting results are shown as dashed lines.

To gain further insights into the structural dynamics of photoactivation, we determined the kinetics for the time-dependent evolution at the wavenumbers of some of the key transients and bleaches in the TRIR spectra (Figure 4). The kinetic traces of the raw data are shown as dots, while the fit recovered from Global analysis is shown by dashed lines. In Figure 4A, the excited state of the flavin, represented by the 1383 cm^–1^ transient, decays more rapidly than the rate of ground-state recovery (1546 cm^–1^ bleach), indicating that relaxation of the excited-state involves intermediate species that precede reformation of the ground state. The intermediate species formed are flavin radicals, and in Figure 4B, we compare the kinetics of the two flavin radical intermediates, FADH^•^ and FAD^•-^, to show that they are both forming at the similar time, indicating that the electron and proton transfer is not sequential, but rather that concerted proton-coupled electron transfer is occurring. Furthermore, we compared the kinetics of FADH^•^ at 1528 cm^–1^ with the transient at 1635 cm^–1^ which demonstrates that both species evolve with the same rate constant (Figure 4C), suggesting that the 1635 cm^–1^ vibrational mode is also marker for the FADH^•^ state of the flavin. In addition, the transient observed at 1694 cm^–1^ (Figure 4D) reflects the formation of the second hydrogen bond to the flavin C4=O group found in the light state with a time constant of 184 ps.

The photocycle of OaPAC can be compared with that observed for other BLUF proteins. For instance, in PixD, the anionic radical (FAD^•-^) transient at 1515 cm^–1^ is formed in 2.5 ps and then starts to decay around 20 ps, followed by the appearance of the FADH^•^ transient at 1535 cm^–1^ with a time constant of 110 ps. Thus, the formation of the two radical species in PixD are well separated in time and formed sequentially following the FAD* → FAD^•-^ → FADH^•^ photocycle, which is also observed in other photoactive flavoproteins such as the cryptochrome superfamily.^35,36^ However, in wild-type OaPAC, we observe a transient at 1520 cm^–1^ which we suggest has contributions from both flavin radicals (FAD^•-^ and FADH^•^). The presence of an FADH^•^ population is supported by the appearance of the transient at ∼1510 cm^–1^ at early times which can be assigned to a neutral tyrosine radical.^33^ Therefore, the FADH^•^-Tyr^•^ radical pair is formed very fast after excitation. This assumption is in harmony with the photoactivation model proposed by Ohki et al.^18^ The presence of the 1520 cm^–1^ transient suggests that there are two possible (parallel) electron transfer routes where the anionic and neutral flavin radical can be formed at the same time. As we discuss later, replacement of Y6 with F-Tyr residues halts the photocycle at FAD^•-^ resulting in a clearly observed transient at 1515 cm^–1^.

### Visible Transient Absorption Spectroscopy

**Figure 5:**
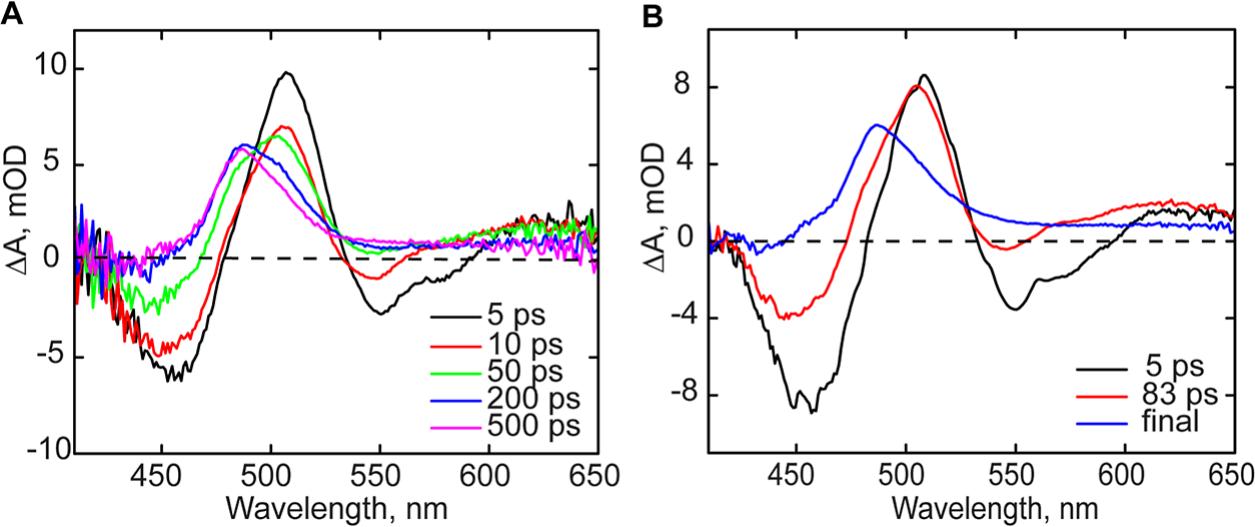
Transient absorption spectra of wild-type OaPAC. (A) Spectra recorded at different time delays. (B) EAS spectra of wild-type OaPAC determined by global analysis.

TA measurements of wild-type OaPAC were also performed in the visible range to obtain additional information on the photoactivation mechanism (Figure 5). Similar to the TRIR spectra, global analysis of the TA measurements reveals three dominant EADS (evolution-associated difference spectra) which have been analyzed by spectral fitting (Figure S2). The first 5 ps EADS can be assigned to the decay of the oxidized flavin excited state (FAD*-FAD_ox_), which evolves to the next EADS (red line) with a lifetime of 83 ps (Figure 5B). Besides the absorption band at ∼505 nm, the second EADS component shows a decrease of the stimulated emission band at 550 nm, partial loss of the ground state bleach and a shoulder at ∼490 nm. EADS2 strongly resembles the spectrum of a neutral semiquinone flavin radical, FADH^•^,^37^ and the spectral fitting reveals substantial contributions from FADH^•^. The non-decaying EADS (final or asymptotic spectrum, blue line), which has been extrapolated to 1 μs, shows the disappearance of the ground-state bleach, and the appearance of an absorption feature near 488 nm. This non-decaying component is modeled as the difference of the light-adapted and dark-adapted linear absorption spectra of wild-type OaPAC.

### Mutagenesis of Y6 and W90 to Investigate the Electron Donor in Wild-Type OaPAC

Photoactivation of OaPAC involves an ultrafast electron transfer to the flavin following excitation (Figure 3). Although the conserved tyrosine (Y6) is thought to be the primary electron donor, the adjacent tryptophan W90 could also function as a competitive source of electrons. To explore this possibility, we constructed the Y6F and W90F OaPAC mutants and characterized their photophysical properties. Phenylalanine is not expected to function as an electron donor to FAD*.^33^ Although the Y6F mutant is photoinactive, irradiating Y6F leads to the fully reduced form of the flavin forming (FADH^–^) (Figure S3). This behavior is different from the Y7F bPAC mutant where the neutral semiquinone is formed upon blue light illumination.^3^ The W90F mutant is however still able to form a light state albeit with a ∼5-fold reduction in the rate of dark-state recovery (Figure S3).

Even though Y6F does not form a (meta) stable light state in the UV–vis measurement, the TRIR data indicate a sequential formation of FAD^•–^ (1518 cm^–1^) followed by FADH^•^ (1528 cm^–1^) (Figure S4). A transient is also observed at 1488 cm^–1^ which is consistent with the presence of a tryptophan cation radical (TrpOH^•+^), suggesting that W90 can act as an electron donor in the absence of Y6.^33^ This is in agreement with studies by Gauden et al. who demonstrated that W104 in AppA can act as an electron donor albeit via a pathway that does not contribute to the light sensing of AppA.^38^ In addition, Ishikita calculated that the redox potential of W104 in the dark and light adapted states is as high as 1.133 and 1.116 V, respectively, assuming a configuration in which the tryptophan is oriented away from the flavin.^39^ Thus, the tryptophan that is found adjacent to the flavin in most BLUF proteins (W104 in AppA, W91 in PixD, W90 in OaPAC) is fully able to function as an electron donor.

The TRIR spectra of Y6F thus show that mutation of the conserved tyrosine does not abolish electron transfer and that the formation of radical species occurs sequentially, as observed in PixD,^29^ but on a substantially longer timescale. First, the formation of the anionic flavin radical (FAD^•–^) at 1518 cm^–1^ occurs in 16 ps, followed by the formation of the neutral (FADH^•^) flavin radical at 1528 cm^–1^ in ∼100 ps (Figure S4). As expected, there is no transient observed at 1694 cm^–1^ as a light-adapted state is not formed in this mutant. The tryptophan (W) at position 90 is expected to be the electron donor because the primary electron donor (Y6) is absent. Indeed, the formation of the 1488 cm^–1^ peak reflects the formation of the tryptophan cation radical (TrpOH^•+^),^33^ which is again consistent with the fact that removing Y6 leaves W90 as the only electron donor. The Y to F mutation was also introduced into PixD (Y8F);^29^ however, unlike OaPAC, the TRIR data show that the Y8F PixD mutant was only able to participate in one transfer process, either electron transfer or proton transfer (PT) because the rise and decay of the signal at 1515 and 1530 cm^–1^ had the same kinetics.^29^

The TRIR spectra of W90F OaPAC reveal the formation of the flavin neutral radical species (FADH^•^) as seen in the wild-type protein (Figure S5). The peak observed at 1512 cm^–1^ reflects the formation of the neutral tyrosine radical as in the case of wild-type OaPAC.^33^ In addition, the W90F mutant contains the same pair of modes observed in the wild-type at 1621 (−)/1631 (+) cm^–1^, as well as the 1693 cm^–1^ the vibrational mode which is associated with the formation of the light state.

TA measurements of W90F mutant were also performed in the visible range and compared to wild-type OaPAC (Figure S6A). Similar to the wild-type, global analysis of the TA measurements reveals three dominant EADS which have been analyzed by spectral fitting (Figure S6B). The first EAS of W90F resembles that observed in wild-type OaPAC and is dominated by the excited state of the flavin and corresponding ground state bleach. A striking difference is observed in the 520–570 nm region of the second EAS where one can see a larger positive feature compared to the wild-type data. Spectral fitting of EAS2 shows that in the case of this mutant, we have a larger contribution from the neutral semiquinone (FADH^•^) (Figure S6C). This suggests that the PCET process is more efficient in the W90F mutant compared with the wild-type as the removal of the adjacent tryptophan eliminates the alternate electron transfer route. This difference between the wild-type and W90F mutant was not obvious in the TRIR data; however, the TA data show that the decay of the excited state at 510 nm for W90F is faster than wild-type, indicating that the PCET process is more efficient in the mutant (Figure S6D).

### Effect of Y6 Fluorination on the Photocycle of OaPAC

Y6 is a strictly conserved residue in all BLUF domain proteins and is critical for photoactivity because replacement of this residue with any other amino acid including phenylalanine yields a photoinactive protein. The role of tyrosine as an electron and/or proton donor is pH dependent, and the phenol p*K*_a_ varies depending on whether tyrosine is reduced (p*K*_red_) or oxidized (p*K*_ox_). For tyrosine in solution, p*K*_red_ and p*K*_ox_ have values of 9.9 and −2, respectively, and above pH 9.9 tyrosine deprotonates to form tyrosinate (Y^–^) so that electron transfer generates the neutral tyrosine radical (Y^•^). At pH values between p*K*_red_ and p*K*_ox_ tyrosine is protonated and the Y^•^/Y redox couple has a pH-dependent potential that increases by 59 mV per pH unit. Finally, if the pH is more acidic than p*K*_ox_, electron transfer will generate the cation radical (Y^•+^).^40^

In order to explore the role of the tyrosine p*K*_a_ on OaPAC photoactivation, we thus replaced Y6 with fluorotyrosine (F-Tyr) analogues to modulate the acidity of the phenol. In contrast to tyrosine (p*K*_a_ 9.9), 2,3,5-F_3_Tyr has a p*K*_a_ of 6.4 and is therefore ∼3000-fold more acidic than tyrosine with a reduction potential (Y^•^/Y^–^) that is ∼200 mV higher. Liquid chromatography–mass spectrometry/mass spectrometry analysis did not detect any native tyrosine in the F-Tyr-substituted proteins, indicating that the F-Tyr content of each variant was ≥99% (Figure S7).

#### Electronic State of *n*-FY6 Variants

**Figure 6:**
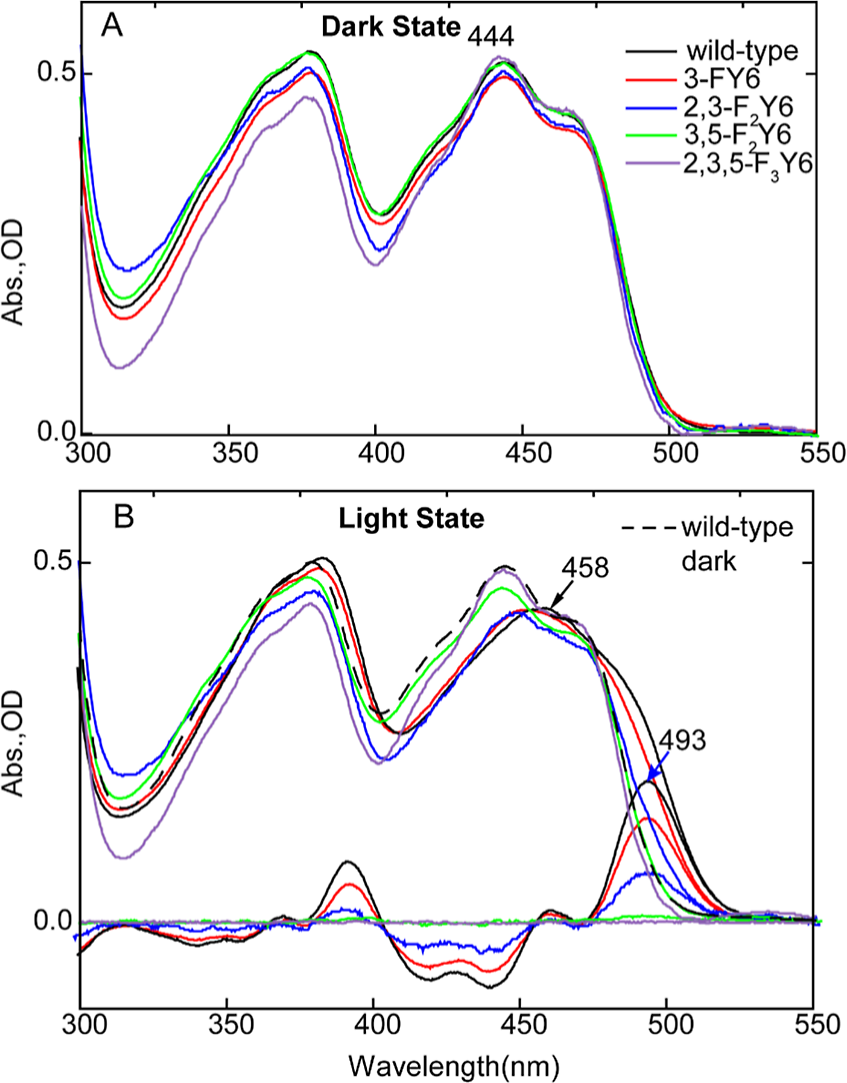
Electronic spectra of *n*-FY6 OaPAC Variants in H_2_O. Comparing the dark (A) and light (B) state absorbance spectra of *n*-FY6 variants to wild-type OaPAC, the flavin electronic state is not perturbed by the fluorotyrosine. Only 3-FY6 and 2,3-F_2_Y6 form a red-shifted spectrum upon illumination of the sample with 450 nm LED, and both have a difference electronic spectrum illustrating an absorbance of the light state at 493 nm.

We first examined the electronic absorption spectrum of the *n*-FY6 OaPAC variants before and after illumination. As observed in Figure 6, all the *n*-FY6 variants have a λ_max_ at 444 nm which is the same as the electronic spectrum of the flavin in wild-type OaPAC, suggesting that the fluorotyrosine analogues have not dramatically perturbed the flavin binding pocket. However, irradiation of each variant only caused a detectable red shift in the flavin absorption for the 3-FY6 and 2,3-F_2_Y6 OaPAC proteins. The shifts were 10 and ∼5 nm, respectively, whereas no red shift was observed for either 3,5-F_2_Y6 or 2,3,5-F_3_Y6 OaPAC within the 10 ms time resolution of the experiment. These results indicate that lowering the p*K*_a_ impacts the stability of the light state.

#### FTIR Steady-State Difference Spectra of the *n*-FY6 OaPAC Variants

**Figure 7:**
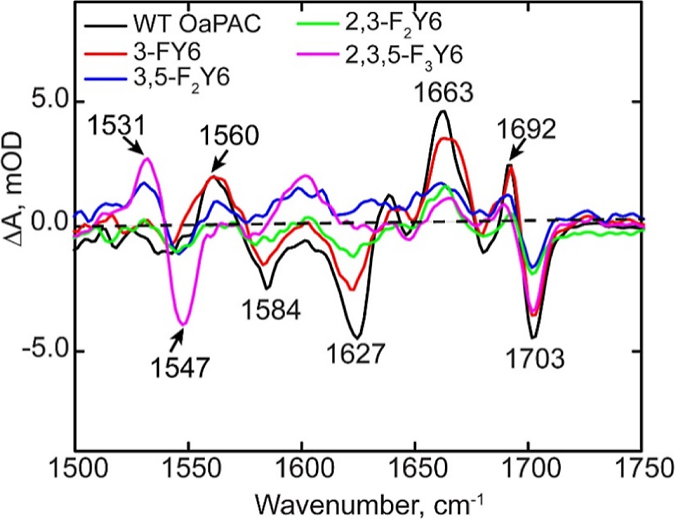
FTIR difference spectra of wild-type OaPAC and *n*-FY6 analogues. Only the L – D difference spectrum of the 3-FY6 resembles the wild-type protein.

The FTIR steady-state difference spectra of each OaPAC *n*-FY6 variant are compared with wild-type OaPAC in Figure 7. Only the difference spectrum of the 3-FY6 variant resembles wild-type OaPAC. Three difference modes at ∼1663 (+) and 1692 (+)/1703 (−) cm^–1^, previously assigned to the C4=O carbonyl of the flavin, are conserved in the FTIR difference spectrum of the 3-FY6 OaPAC. The bleaches at 1584 and 1627 cm^–1^ are also observed which are assigned to protein backbone modes. However, the magnitude of the light-induced structural change is smaller than observed in the wild-type photoreceptor.

The FTIR difference spectra of the 2,3-F_2_Y, 3,5-F_2_Y, and 2,3,5-F_3_Y variants differ the most from wild-type OaPAC in the region of 1500–1650 cm^–1^. Although a small change can be observed around the C4=O carbonyl of flavin (1694 (+)/1703 (−) cm^–1^), only minimal changes are observed in the protein backbone marker modes in the 1600 cm^–1^ region. Compared to 3,5-F_2_Y, and 2,3,5-F_3_Y variants, the difference spectrum of 2,3-F_2_Y6 variant shows the most light-induced changes in the protein backbone modes (1584 and 1627 cm^–1^). Interestingly, the 2,3,5-F_3_Y6 sample has two modes at 1531 (−)/1547 (+) cm^–1^ that are not observed in the wild-type protein but are found in the other *n*-FY6 analogues, albeit with lower intensity.

#### Analysis of *n*-FY6 OaPAC Photoactivation Using TRIR and TRMPS

**Figure 8:**
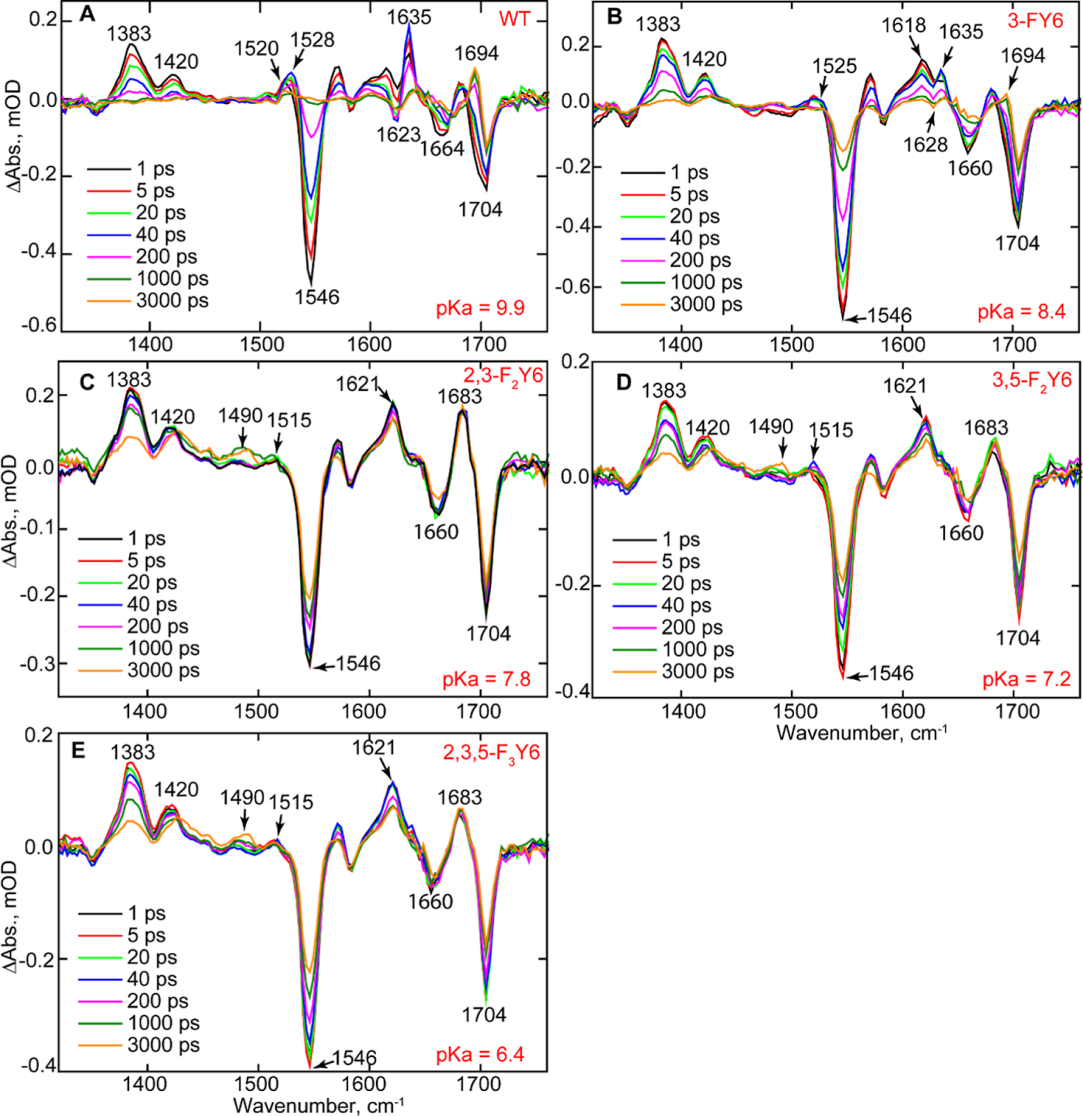
TRIR of wild-type OaPAC and the *n*-FY6 OaPAC variants. Spectra recorded at 1, 5, 20, 40, 200, 1000, and 3000 ps are shown in the figure. (A) Wild-type (WT), (B) 3-FY6, (C) 2,3-F_2_Y6, (D) 3,5-F_2_Y6, and (E) 2,3,5-F_3_Y6.

The forward photoactivation reaction of the *n*-FY6 OaPAC variants was studied using ultrafast TRIR and TRMPS. Figure 8 depicts the temporal evolution of 3-FY6, 2,3-F_2_Y6, 3,5-F_2_Y6, and 2,3,5-F_3_Y6 OaPAC variants. At 1 ps, all the variants contain the same transients and bleaches as the wild-type protein. For instance, transients assigned to the excited state of FAD are observed at 1383 and 1420 cm^–1^, while bleaches at 1546, ∼1660, and 1704 cm^–1^ are also present, which are assigned to the ground state of FAD, C2=O, and C4=O, respectively. However, as the TRIR spectra evolve over time, significant differences are observed compared to the wild-type protein. The TRIR spectra of 3-FY6 OaPAC most closely resemble the wild-type spectrum, although the evolution of the transients and bleaches occurs fivefold slower. Formation of the radical intermediate FADH^•^ is observed at 1525 cm^–1^ in 3-FY6 OaPAC, suggesting that this variant is still able to participate in PCET. However, the amplitude of the radical signal is smaller in 3-FY6 OaPAC. In addition, the differential line shape at 1623 (−)/1635 (+) cm^–1^, observed in wild-type OaPAC is not as pronounced in the 3-FY6 mutant and the bleach is shifted by 5 cm^–1^ from 1623 to 1628 cm^–1^. Finally, the light state, characterized by the transient at 1694 cm^–1^, forms with a time constant of 510 ps, which is much slower than in wild-type OaPAC (184 ps) (Figure S8).

The photocycle of the other *n*-FY6 variants, 2,3-F_2_Y6, 3,5-F_2_Y6, and 2,3,5-F_3_Y6, does not proceed beyond FAD^•^ (transient observed at 1515 cm^–1^), and thus, no transients are observed at 1528 cm^–1^ for FADH^•^ and at 1694 cm^–1^ for the light state (Figure 8). In addition, these variants lack the 1623 (−)/1635 (+) cm^–1^ modes observed in wild-type and 3-FY6 OaPAC. The kinetics of the excited state decay at 1383 cm^–1^ and ground-state recovery at 1546 cm^–1^ are much slower compared with wild-type OaPAC (Table 1). Finally, the TRIR data contain a feature around 1490 cm^–1^, indicative of triplet state formation which is absent in the wild-type OaPAC.

Time-resolved multiple probe spectroscopy (TRMPS) spectra were also recorded for all the *n*-FY6 variants to investigate whether further evolution occurs beyond the timescale of TRIR (3 ns) (Figure S9). The TRMPS data confirm that only 3F-Y6 OaPAC forms a light state, whereas the 2,3-F_2_Y6, 3,5-F_2_Y6, and 2,3,5-F_3_Y6 OaPAC do not have an observable or detectable transient at 1694 cm^–1^. In addition, the transient at 1490 cm^–1^, assigned to a triplet state mode of the flavin, is more distinguishable in all *n*-FY6 variants compared with wild-type where no transient is observed.

### Adenylate Cyclase Activity

An enzymatic assay was performed to examine the impact of modulating the p*K*_a_ and/or reduction potential of Y6 on the ability of OaPAC to convert ATP into cAMP. The formation of pyrophosphate (PPi) was monitored in both discontinuous and continuous formats using a coupled assay based on a PPi-dependent phosphofructokinase pyrophosphate reagent kit that results in the oxidation of NADH, which is monitored at 340 nm.^41,42^ Using a discontinuous assay format in which the amount of PPi was quantified after the reaction was quenched by heating, only the 3-FY6 and 2,3-F_2_Y6 variants had observable activity, whereas 3,5-F_2_Y6 and 2,3,5-F_3_Y6 had no detectable activity (Figure S10). Fitting the data to the Michaelis–Menten equation provided k_cat_/K_M_ values for 3-FY6 and 2,3-F_2_Y6 OaPAC that were comparable with the wild-type (Table S1). Finally, in contrast to the other variants, 2,3-F_2_Y6 had residual activity in the absence of blue light, indicating that a population of this variant is in a pseudo-lit state which is able to covert ATP into cAMP in the absence of light.

**Figure 9:**
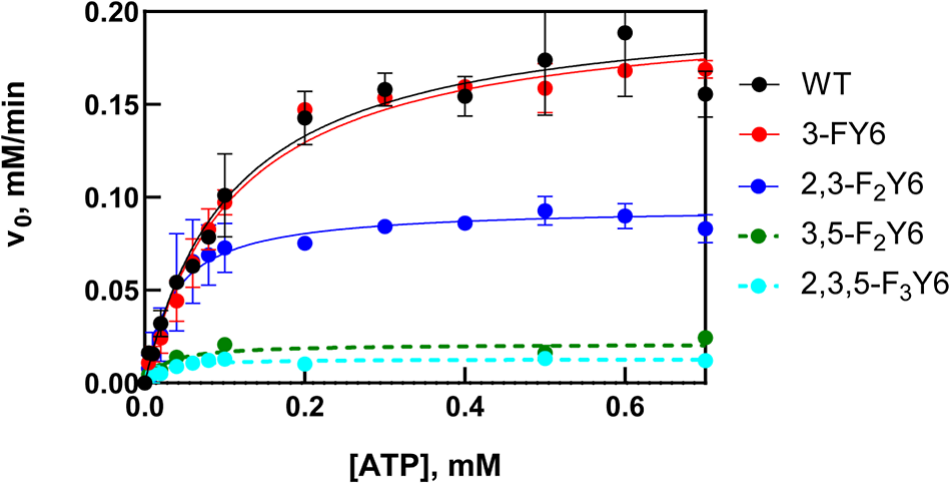
Michaelis–Menten plots of wild-type (WT) OaPAC and *n*-FY6 variants. Each point represents the initial velocity at each ATP concentration extracted from the linear portion of the *A*_340_ vs time plot under continuous illumination. The lines are the result of nonlinear fits to the Michaelis–Menten equation. Each data point is the average of two replicates where the error bars represent the standard error of the mean.

The consumption of NADH as function of time was also monitored in a continuous assay format in which the coupled assay reagents were present during OaPAC photoactivation. Using this method, similar results were obtained for all the variants (Figure 9 and Table 2).

**Table 2:** Kinetic Parameters for Wild-Type OaPAC and *n*-FY6 Variants^a^

	p*K*_a_	*k*_cat_ (min^-^1)	*K*_M_ (mM)	*k*_cat_/*K*_M_ (mM^–1^ min^–1^)
wild-type OaPAC	9.9	205 ± 11	0.12 ± 0.01	1888 ± 207
3F-Y6	8.4	202 ± 6	0.11 ± 0.01	1796 ± 182
2,3-F_2_Y6	7.8	95 ± 5	0.04 ± 0.01	2664 ± 551
3,5-F_2_Y6	7.2	b	b	b
2,3,5-F_2_Y6	6.4	b	b	b

a Data were obtained under continuous illumination using 1 μM enzyme.

b No enzymatic activity detected.

## Discussion

Although the photochemistry of BLUF photoreceptors has been extensively studied using a variety of time-resolved approaches, these studies have been largely confined to BLUF domain proteins lacking the biologically relevant output partner.^23,29,43−45^ In the present work, we extend our analysis of the BLUF photocycle to OaPAC in which both the BLUF and adenylate cyclase output domains are contained in a single protein. Using TRIR and TRMPS, we investigated the OaPAC photoactivation mechanism, and compared it with other BLUF proteins. We further explored the impact of modulating the acidity of the conserved tyrosine Y6 on the light-controlled adenylate cyclase reaction. The photoactivation of the BLUF photoreceptors can be broadly distinguished based on the presence or absence of radical intermediates during light-state formation. Whereas no radical intermediates can be observed during light-state formation in AppA, BlrB and BlsA, the photoactivation of PixD (Slr1694) and PapB involves PCET on the reaction pathway leading to the light state.^23,26,46,47^ In the case of PixD, photoactivation of the flavin leads to a sequential formation of anionic (FAD^•-^) and neutral (FADH^•^) flavin radicals, while in PapB, a neutral flavin semiquinone FADH radical (FADH^•^) was observed as the intermediate before the formation of the signaling state.^29,46^

The TRIR and visible TA data indicate that two different processes can occur in the OaPAC photocycle after excitation. TRIR measurements on the wild-type protein and the Y6F mutant illustrate that if W90 is present, there is an electron transfer process from W90 to the flavin, forming the FAD^•-^ TrpOH^•+^ radical pair. TRIR and visible transient measurements on the wild-type and W90F mutant also indicate that after excitation, a concerted proton-coupled electron transfer process occurs from the Y6 to the flavin, generating the FADH^•^–Tyr^•^ radical pair.

The role of proton transfer in the function of OaPAC_BLUF_ was examined in a recent paper by the Zhong group where the authors replaced the tyrosine by a tryptophan (Y6W).^48^ Kang et al. observed a sequential electron transfer process: formation of the flavin anionic radical, followed by the formation of the neutral semiquinone. The authors proposed that they observed proton rocking in which the tryptophan transiently donated a proton to the flavin to form the neutral semiquinone followed by a reverse PT; the rates of the forward and reverse PT were very close, being 51 ps and 52 ps, respectively. We have observed enhancement of the electron transfer process in other BLUF domain proteins (AppA, PixD) when the conserved tyrosine (Y21 and Y8, respectively) was replaced with a tryptophan, although this mutation resulted in the loss of protein activity. We expect that a similar effect occurs in the case of the Y6W OaPAC mutant and plan to observe the effect of this mutation on the cAMP production.

The conserved tyrosine in BLUF domain proteins is essential for photoactivity, and in every case, including OaPAC, replacement of this residue with phenylalanine results in a photoinactive protein. However, the precise role of the conserved tyrosine in the photoactivation mechanism depends on whether or not radical intermediates are present in the photocycle. In PixD, the 3000-fold increase in acidity of Y8 resulting from replacing Y8 with 2,3,5-F_3_Y6 halts the photocycle at FAD^•-^ presumably because the tyrosine is ionized and can no longer function as the proton donor required for the formation of FADH^•^. In contrast, replacement of Y21 in AppA has only a slight impact on the kinetics of light-state formation and every *n*-FY6 variant is photoactive. The biggest alteration in AppA_BLUF_ was on the dark-state recovery where the change in Y21 p*K*_a_ led to a 4000-fold increase in the rate of dark-state recovery in H_2_O, while in PixD the change was only 15-fold.^29^ The studies here show again that OaPAC photochemistry resembles PixD. For 3,5-F_2_Y6 and 2,3,5-F_3_Y6, the two variants with the most acidic phenol groups (p*K*_a_ 7.2 and 6.4), no light state can be observed in either the absorption spectrum or the TRIR spectrum. In contrast for 3-FY6, where the phenol p*K*_a_ is only 1.5 pH units more acidic than tyrosine, a 10 nm red shift is observed in the flavin absorption spectrum upon excitation and the TRIR/TRMPS spectra are very similar to wild-type OaPAC. Finally, although a ∼5 nm red shift in the flavin absorbance at 450 nm can be observed upon irradiation of 2,3-F_2_Y6 OaPAC, no light-state transient at 1694 cm^–1^ can be observed in the TRIR and the photocycle apparently stalls at FAD^•-^. We speculate that the lack of observable light state in the TRIR data is because the yield of light state is low in this mutant given that this is a single shot experiment.

The presence of a covalently attached adenylate cyclase domain in OaPAC provides a unique opportunity to directly link the photochemistry of the BLUF domain with activation of the output domain. Using a coupled assay, light-dependent conversion of ATP into cAMP occurs with *k*_cat_, *K*_M_, and *k*_cat_/*K*_M_ values of 205 ± 11 min^–1^, 0.12 ± 0.01 mM, and 1888 mM^–1^ min^–1^, respectively. These values have not been previously reported for OaPAC; however, Ohki et al. reported an ∼20-fold change in enzymatic activity between dark and light states at a single ATP concentration.^7^ In the pyrophosphate spectrophotometric assay, we observe a ∼100-fold increase in activity for wild-type OaPAC upon photoexcitation. In agreement with time-resolved spectroscopy, 3-FY6 OaPAC has adenylate cyclase activity that is comparable to that of the wild-type protein, whereas 3,5-FY6 and 2,3,5-FY6 show no light-dependent catalytic activity. Interestingly, 2,3-F_2_Y6 has a similar *k*_cat_/*K*_M_ value to wild-type OaPAC even though no light state can be observed in the TRIR spectrum and the steady-state FTIR difference spectrum showed smaller light-induced changes in the protein modes in contrast to the wild-type. As noted above, a small red shift can be observed in the flavin absorption band at 450 nm, suggesting that light state can be formed when the protein is continuously illuminated. We speculate that in the assay, we are converting all the photoactive 2,3-FY6 into the light state, whereas there is only a low yield of light state in the single-shot TRIR experiments (see above). Also, a population of 2,3-F_2_Y6 variant is in a pseudo-lit state because in the assay, we observed conversion of ATP to cAMP in the absence of light. Therefore, in the case of the steady-state IR difference measurement, the appearance of protein modes should appear suppressed compared to the wild-type (see above).

## Conclusions

Our time-resolved experiments on BLUF domain photoreceptors have been extended to OaPAC in which the BLUF domain is covalently attached to an AC output domain. OaPAC is thus a good model system not only for studying photochemistry but also for elucidating signal transduction between a BLUF domain and an output domain by monitoring the light-stimulated conversion of ATP into cAMP. Our work illustrates the direct impact of the photochemical processes in the BLUF domain on the output domain, which has not previously been shown in AppA, PixD, or any other BLUF domain. Using ultrafast infrared and transient absorption spectroscopy, we show that the photoactivation mechanism of OaPAC involves concerted proton electron transfer from the conserved Y6 to the excited state of FAD (FAD*). This mechanism is slightly different from the one observed in PixD, where the photoactivation mechanism involves a sequential proton-coupled electron transfer from Y8 to FAD*. Instead, in the case of OaPAC protonation of the flavin occurs together with the electron transfer step from Y6 to the flavin. The role of Y6 in the photocycle of OaPAC was probed via UAA mutagenesis. Replacement of Y6 with *n*-FY analogues increases the acidity of the phenol hydroxyl group and reduces the rate of electron transfer. Specifically, altering the p*K*_a_ and/or reduction potential of the flavin had a profound impact on light-state formation for 2,3-F_2_Y6, 3,5-F_2_Y6, and 2,3,5-F_3_Y6 where the photocycle is halted at FAD^•-^. Using an enzyme assay that couples PPi production to the consumption of NADH, we quantified the adenylate cyclase activity of wild-type OaPAC and also of the *n*-FY6 variants to interrogate the impact of the Y6 p*K*_a_ on the light-activated conversion of ATP into cAMP and PPi. Only the *n*-FY6 variants with p*K*_a_ values of 7.8 or higher were able to catalyze cAMP formation, while variants with lower p*K*_a_ values were inactive because the photocycle was halted at FAD^•-^. While the 2,3-F_2_Y6 OaPAC variant (p*K*_a_ 7.8) had light-dependent adenylate cyclase activity, no light state was observed in the TRIR which we propose is due to the low yield of product formation given the single-shot format of the experiment. Collectively, the results shed new light on the photoactivation mechanism of BLUF domain photoreceptors.

## Supporting Information Available

Experimental procedures including spectroscopic methods and the synthesis and incorporation of fluorotyrosine residues; kinetic parameters for OaPAC; IR, TRIR, transient absorption, and UV–vis spectra; MALDI spectra of F-Tyr containing peptides; and Michaelis–Menten plots of enzyme activity (PDF)

## Abbreviations

BLUF: blue light using flavin adenine dinucleotide
cAMP: cyclic adenosine monophosphate
EADS: evolution-associated difference spectra
OaPAC: *Oscillatoria Acuminata* photoactivated adenylyl cyclase
OaPAC_BLUF_: BLUF domain of OaPAC
OaPAC FL: full length OaPAC
PAC: photoactivated adenylyl cyclase
PCET: proton coupled electron transfer
PT: proton transfer
TRIR: time-resolved infrared
TRMPS: time-resolved multiple probe spectroscopy
UAA: unnatural amino acid
