# Supplementary material for: Unraveling the Photoactivation Mechanism of a Light-Activated Adenylyl Cyclase Using Ultrafast Spectroscopy Coupled with Unnatural Amino Acid Mutagenesis
Source: ACS Chem Biol. 2022 Aug 29;17(9):2643–54. doi: 10.1021/acschembio.2c00575 (PMC9486806; doi:10.1021/acschembio.2c00575)
Supplement: Supplementary file 1 — cb2c00575_si_001.pdf [file cb2c00575_si_001.pdf]

Supporting Information

Unraveling the Photoactivation Mechanism of a Light Activated Adenylyl Cyclase using Ultrafast Spectroscopy coupled with Unnatural Amino Acid Mutagenesis

Jinnette Tolentino Collado,[†] James N. Iuliano,[†] Katalin Pirisi,[§] Samruddhi Jewlikar,[†] Katrin Adamczyk,[‡] Gregory M. Greetham,[‖] Michael Towrie,[‖] Jeremy R. H. Tame,[#] Stephen R. Meech,[‡,]* Peter J. Tonge,[†,]* and Andras Lukacs*[,§]

[†]*Department of Chemistry, Stony Brook University, New York, 11794, United States.* [§]*Department of Biophysics, Medical School, University of Pecs, Szigeti str. 12, 7624 Pecs, Hungary.* [‡]*School of Chemistry, University of East Anglia, Norwich, NR4 7TJ, U.K.* [‖]*Central Laser Facility, Research Complex at Harwell, Rutherford Appleton Laboratory, Didcot, OX11 0QX, U.K.* [#]*Drug Design Laboratory, Graduate School of Medical Life Science, Yokohama City University, Tsurumi, Yokohama, 230-0045, Japan.*

*Authors to whom correspondence should be addressed: Email: s.meech@uea.ac.uk (SRM); peter.tonge@stonybrook.edu (PJT); andras.lukacs@aok.pte.hu (AL)

Table of Contents

Table S1: Kinetic parameters for OaPAC using the discontinuous assay. S3

Figure S1: IR Difference Spectra of Wild-type OaPAC. S4

Figure S2: Visible transient absorption spectra. S5

Figure S3: UV-vis spectra of W90F and Y6F OaPAC mutants. S6

Figure S4: TRIR data of Y6F. S7

Figure S5: Transient infrared spectra of the W90F mutant at different time delays. S8

Figure S6: Transient absorption spectra of W90F OaPAC. S9

Figure S7: Mass spectrometric analysis of F-Tyr incorporation into OaPAC. S10

Figure S8: Global analysis of the TRIR data for the F-Tyr OaPAC variants. S11

Figure S9: TRMPS Spectra of wild-type and F-Tyr OaPACs. S12

Figure S10: Michaelis-Menten plots for wild-type and n-FY6 OaPAC. S13

Experimental Procedures: S14-S18

References: S19

Table S1. Kinetic parameters for OaPAC using the discontinuous assay.

	pKa	k_{cat} (min^{-1})	K_M (mM)	k_{cat}/K_M (mM^{-1} min^{-1})
wild-type OaPAC	9.9	158 ± 7	0.10 ± 0.01	1557 ± 207
3F-Y6	8.4	146 ± 18	0.12 ± 0.04	1243 ± 471
2,3-F_2Y6	7.8	115 ± 12	0.07 ± 0.02	1655 ± 603
3,5-F_2Y6	7.2	***	***	***
2,3,5-F_3Y6	6.4	***	***	***

*** No Enzymatic activity detected.

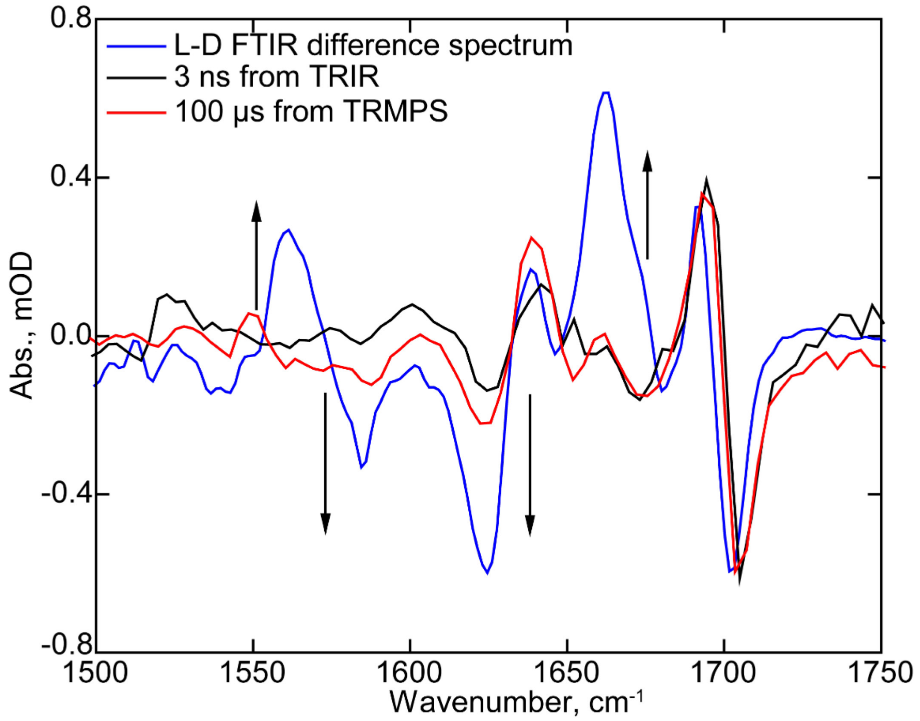

Figure S1. IR difference spectra of wild-type OaPAC. Comparison of difference spectra obtained at 3 ns from TRIR (black), 100 µs from TRMPS (red) and steady state FTIR spectroscopy (blue). We observed further evolution of the protein in 1550-1650 cm^{-1} region.

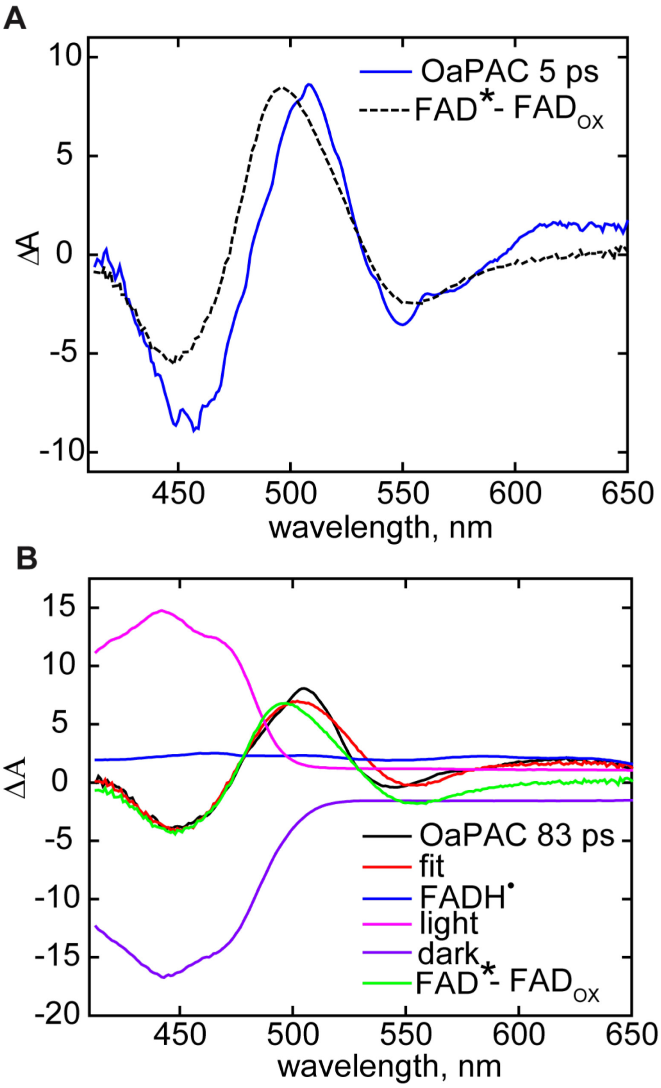

Figure S2. **Visible transient absorption spectra.** Evolution associated spectra analyzed by spectral fitting. A. 5 ps EAS component was fitted with the (FAD*-FAD$_{ox}$) transient absorption spectrum of FAD. B. 83 ps EAS component for WT-OaPAC was fitted as the linear combination of (light-dark)+ FADH$^{\circ}$+ (FAD*-FAD$_{ox}$) spectra.

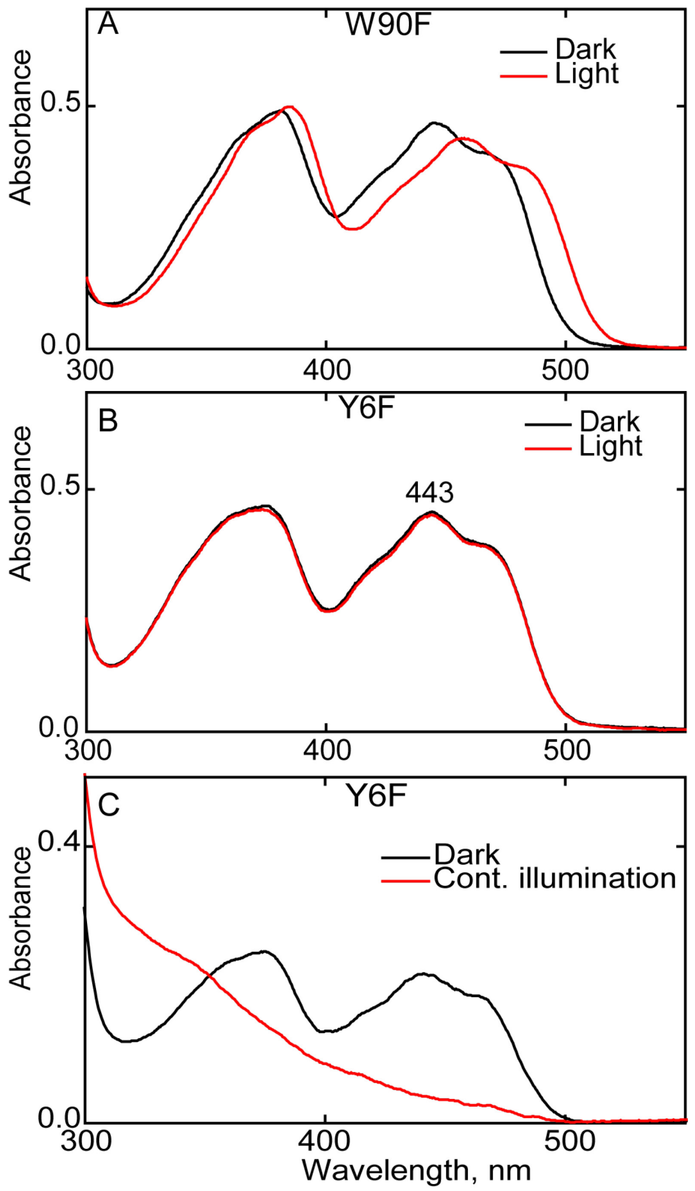

Figure S3. UV-vis spectra of W90F and Y6F OaPAC mutants. A.W90F forms a red-shifted spectrum following 30 s illumination with a blue LED. B.Y6F mutant does not form a red-shifted spectrum following 30 s illumination and hence is photoinactive. C. Continuously illuminating Y6F mutant for 10 min fully reduces the flavin to $FADH^-$.

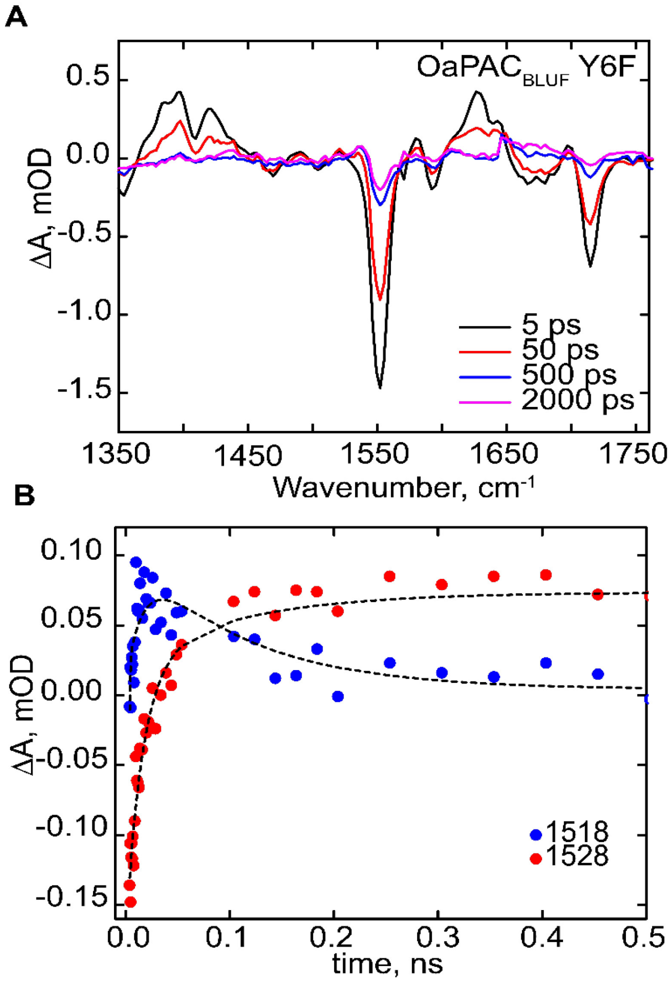

Figure S4. TRIR data of Y6F. A) Transient infrared spectra of the Y6F mutant at different time delays. B) Kinetic traces showing the rise and decay of FAD$^{\bullet-}$ and FADH$^{\bullet}$ in the Y6F mutant.

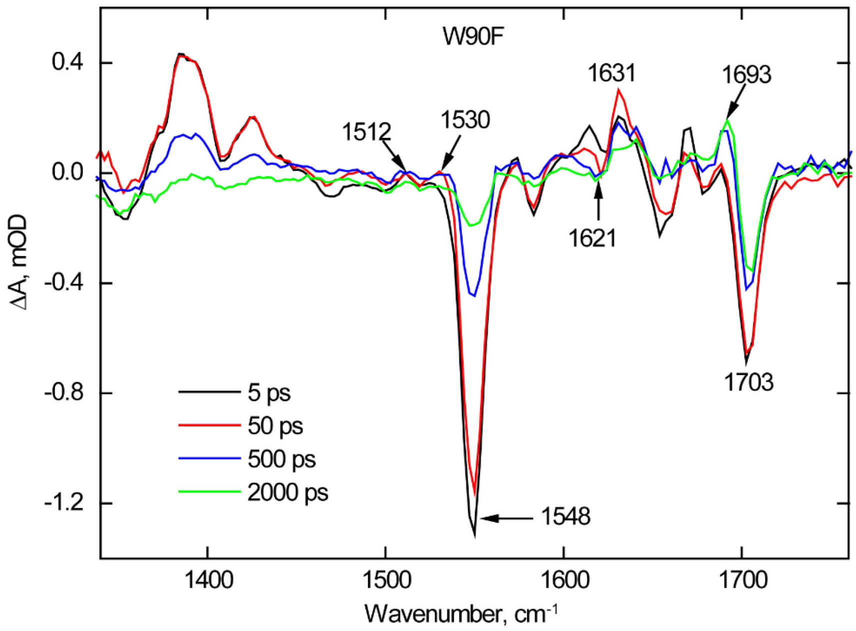

Figure S5. Transient infrared spectra of the W90F mutant at different time delays. The TRIR spectra of the W90F mutant illustrate the formation of the flavin neutral radical (FADH•). The peak formed at ~ 1512 cm^{-1} can be assigned as the tyrosine neutral radical (Tyr•). Also, the W90F mutant contains the same pair of modes observed in the wild-type at 1621 (-) / 1631 (+) cm^{-1}, and 1693 cm^{-1}, the vibrational peak associated to the formation of the light state.

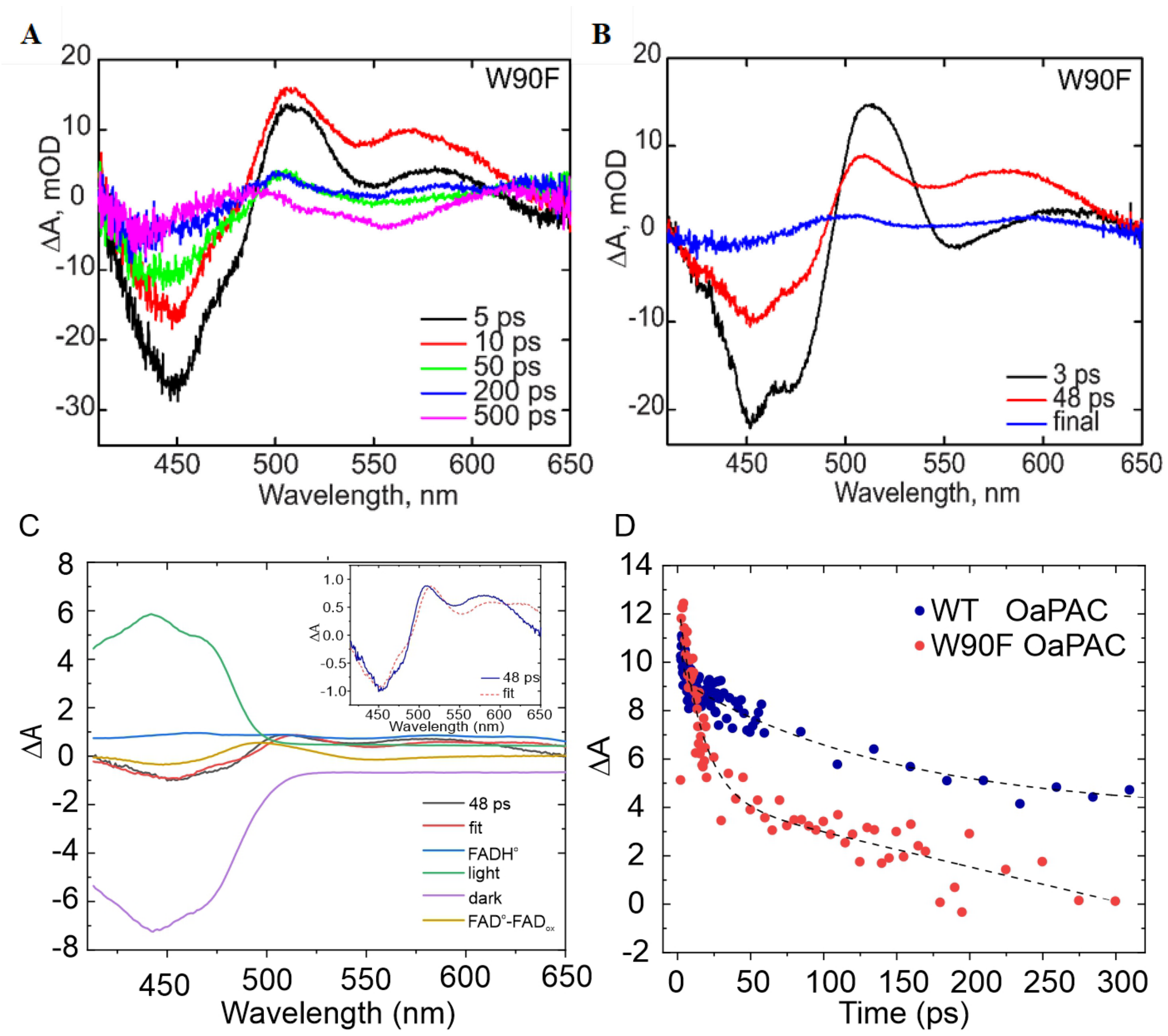

Figure S6. Transient absorption spectra of W90F OaPAC. A) TA of W90F at different time delays B. EAS spectra of W90F OaPAC determined by global analysis. C. Spectral fitting of the 48 ps component of W90F; the inset shows the EAS and the fitted spectrum enlarged. D) Excited state decay observed at 510 nm in the case of wild-type (WT, blue) and W90F OaPAC (red).

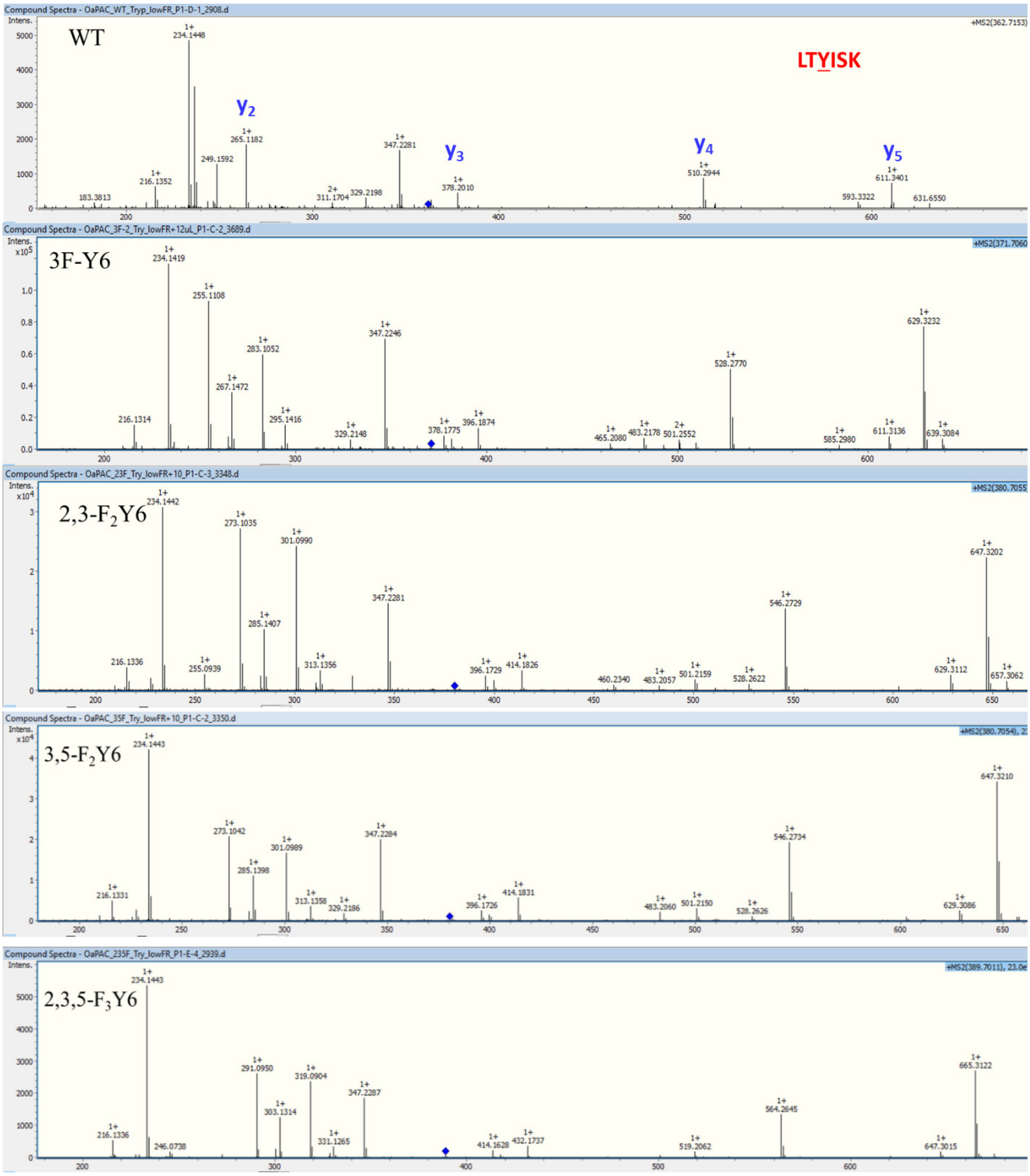

Figure S7: **Mass spectrometric analysis of F-Tyr incorporation into OaPAC.** LC-MS/MS mass spectra were acquired using a Bruker Impact II quadrupole time-of-flight (QTOF, Bruker Daltonics, Billerica, MA, USA) . The mass spectra are for the peptide that contains residue 6: LTY*ISK. In each case no precursor masses were identified for peptides containing the unmodified Tyr.

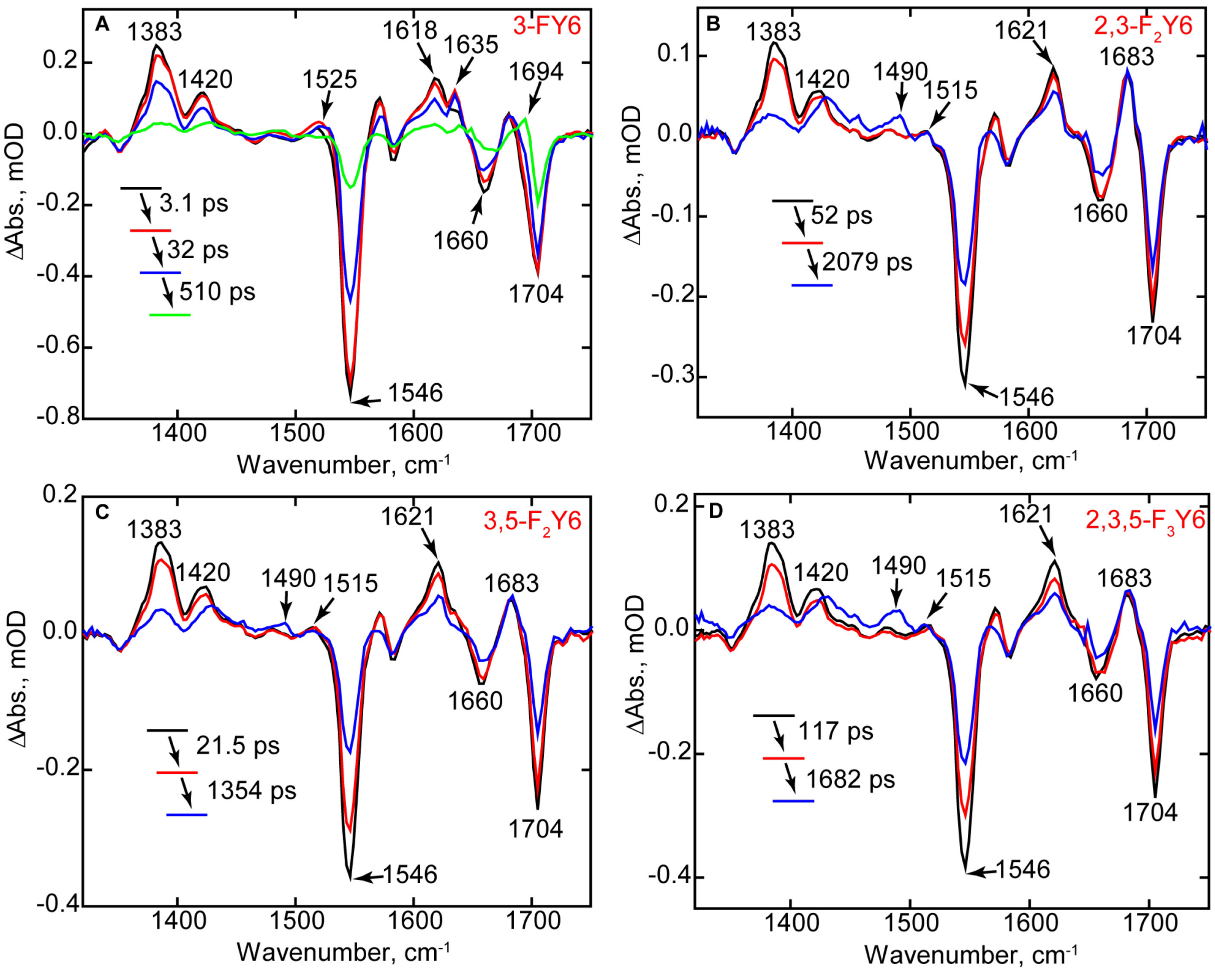

Figure S8: Global analysis of the TRIR data for the F-Tyr OaPAC variants. (A) 3-FY6 OaPAC, (B) 2,3-F_2Y6 OaPAC, (C) 3,5- F_2Y6 OaPAC and (D) 2,3,5-F_3Y6 OaPAC.

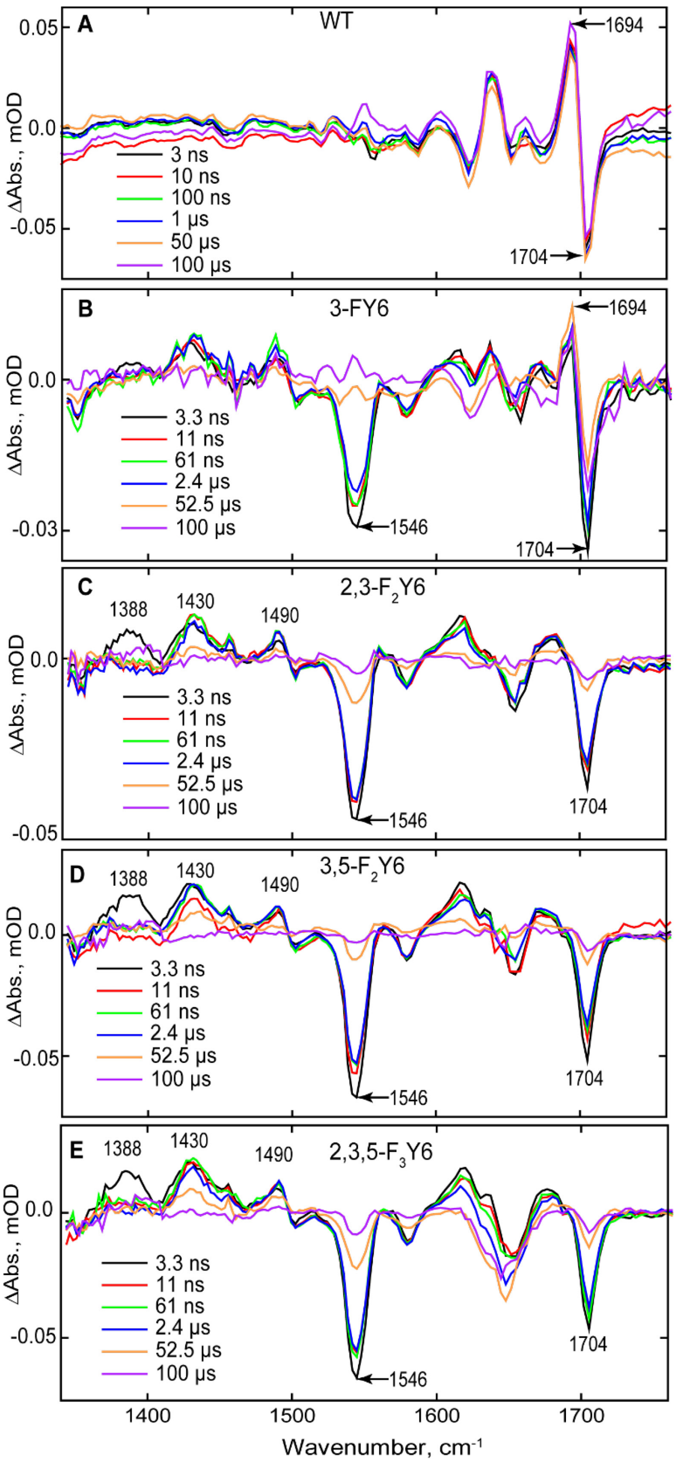

Figure S9. **TRMPS Spectra of wild-type (WT) and F-Tyr OaPACs** (A) WT OaPAC, (B) 3-FY6 OaPAC, (C) 2,3-F_2Y6 OaPAC, (D) 3,5-F_2Y6 OaPAC and (E) 2,3,5-F3Y6 OaPAC. There is no further evolution observed of the transient at 1694 cm^{-1} for WT OaPAC and 3FY6 mutant. For the 2,3-F_2Y6, 3,5-F_2Y6 and 2,3,5-F_3Y6 OaPAC variants, the 1690 cm^{-1} transient is not observed even on the µs timescale. Transients are observed at 1430 and 1490 cm^{-1}, which are indicative of triplet state formation consistent with an increase in lifetime of the excited state.

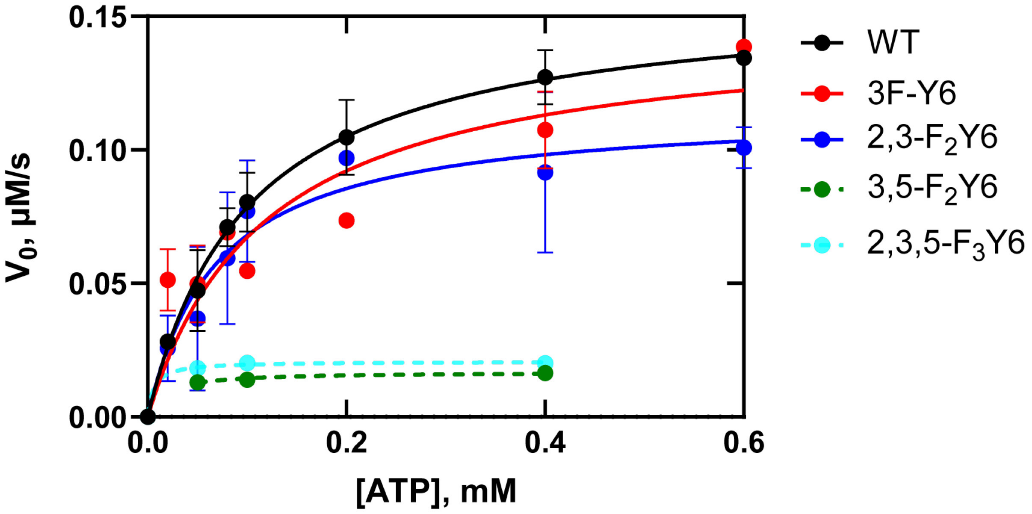

Figure S10. Michaelis-Menten plots for wild-type (WT) and n-FY6 OaPAC. Data were obtained using the discontinuous assay under constant illumination.

Experimental Procedures:

Materials

2-Fluorophenol, 3-fluorophenol and 2,6-difluorophenol were purchased from Sigma-Aldrich. 2,3-Difluorophenol was purchased from Acros Organics. 2,3,6-Trifluorophenol was purchased from Oakwood Chemical. Pyridoxal-5'-phosphate and flavin adenine dinucleotide and purchased from Sigma-Aldrich. The two orthogonal polyspecific aminoacyl-tRNA synthetases, E3 and E11, used to incorporate n-FY analogues were generously provided by Prof. Stubbe, MIT.

Fluorotyrosine Synthesis using Tyrosine Phenol Lyase (TPL)

The expression and purification of TPL, and the synthesis of n-FY analogues were performed using the method previously reported.[1] For this work, only the 3,5-F_2Y6 and 2,3,5-F_3Y6 were synthesized and purified. Briefly, TPL was expressed and purified from BL21 (DE3) *pLysE Escherichia coli* (*E. coli*), and then added to reaction mixtures containing 60 mM sodium pyruvate, 40 μM pyridoxal-5′-phosphate, 30 mM ammonium acetate, 5 mM β-mercaptoethanol and 10 mM the appropriate phenol. After 4 days, the fluorotyrosine analogues were purified, characterized using NMR and mass spectrometry, and lyophilized.

Expression and Purification of Full-Length and BLUF OaPAC

The pCold-I-OaPAC plasmid was transformed into Bl21(DE3) *E. coli* and grown on an LB-agar plate containing 100 μg/mL ampicillin. A single colony was used to inoculate 10 mL 2x-YT media (Fisher Bioreagents, BP9743-5) containing 100 μg/mL ampicillin which was shaken overnight at 37 °C (250 RPM). Subsequently, the 10 mL culture was used to inoculate 1 L of 2x-YT media in a 4 L flask. The culture was shaken at 37 °C (250 RPM) until the optical density (OD_{600}) reached ~0.8-1. The temperature was lowered to 18 °C, and after 30 min protein expression was induced by adding 0.7 mM IPTG. The cells were harvested after 18 h of induction by centrifugation and the cell pellet was stored at -20 °C until needed. The same protocol was performed for the OaPAC BLUF domain.

The cell pellet containing wild-type OaPAC was thawed and resuspended in resuspension buffer (50 mM NaH_2PO_4 pH 8.0, 300 mM NaCl). Subsequently, 14 μL of β-mercaptoethanol, phenylmethylsulphonyl fluoride (PMSF, 250 μM final concentration), and ~10 mg of FAD were added, and the resuspended cells were disrupted using a French Press (Constant Systems Cell Disruptor) at a pressure of 30 kPSI and at 4 °C. The cell debris was removed by centrifugation at 40,000 RPM (185,511 x g) for 1 h at 4 °C, and the supernatant was loaded on to a Ni-NTA column

(25 mL containing 5 mL of resin) equilibrated with resuspension buffer. The column was washed sequentially with 100 mL of resuspension buffer containing 0, 5, 10, 20, and 30 mM imidazole, and OaPAC was then eluted using resuspension buffer containing 250 mM imidazole. Fractions containing protein were pooled and immediately exchanged into 20 mM Tris 150 mM NaCl pH 8.0 using a Bioscale P-6 desalting column. Only the protein samples for TRIR measurements were flash frozen with liquid nitrogen (or liquid N_2) and lyophilized. The protein for the assay was used immediately after purification.

Incorporation of Fluorotyrosines using Orthogonal Aminoacyl-tRNA Synthetases

To extend our study to fluorotyrosine analogues with lower pK_a values, 3-FY, 2,3-F_2Y, 3,5-F_2Y and 2,3,5-F_3Y were incorporated into position 6 of full-length OaPAC. These analogues are not recognized by tyrosyl–tRNA synthetase, and instead were introduced into the protein using E3 and E11, two orthogonal polyspecific aminoacyl-tRNA synthetases. Site-directed mutagenesis was first used to generate a plasmid in which the codon for Y6 was replaced by the TAG stop codon (pCold-I-OaPAC Y6TAG) using pCold OaPAC as template, and the following primers:
5'-GCATATGAAACGCTTAACGtagATCAGCAAATTTTCTCG-3' (forward)
5'- CGAGAAAATTTGCTGATctaCGTTAAGCGTTTCATATGC-3 (reversed). The mutagenesis was verified by the DNA sequencing performed by Genomic Core Facility at Stony Brook University.

The pCold-I-OaPAC Y6TAG plasmid was then co-transformed into BL21(AI) *E. coli* cells together with either the E3 (for 3-FY, 3,5-F_2Y and 2,3,5-F_3Y) or E11 (2,3-F_2Y) pEVOL plasmid and plated on LB-agar containing both 100 μg/mL ampicillin and 34 μg/mL chloramphenicol to select for cells harboring both plasmids. Subsequently, a single colony was used to inoculate 10 mL of 2xYT media containing 100 μg/mL ampicillin and 34 μg/mL chloramphenicol which was incubated overnight at 37 °C (250 rpm) before 5 mL was then used to inoculate 500 mL of the same media. After 2 h of incubation, 0.05% w/v arabinose was added to induce the expression of the E3 or E11 synthetase. The culture was grown until the OD_{600} reached ~ 0.4, and 110 mg of the fluorotyrosine analogue in powder form was added to the media to give a final concentration of ~1 mM. The culture was incubated at 37 °C (250 rpm) until the OD_{600} reached ~1.0, and then the temperature was lowered to 18 °C. After 30 min, 0.7 mM IPTG was added to the media and the cells were harvested after overnight incubation at 18 °C (250 rpm). The n-FY6 samples were purified using the same protocol as that described for the wild-type protein (above). The incorporation of n-FY6 analogue was verified by LC-MS/MS mass spectroscopy.

Light – Dark Steady- State FTIR Difference Spectra

The steady-state spectra were obtained on a Vertex 80v (Bruker) FTIR spectrometer with 3 cm^{-1} resolution and 256 scans. Measured protein samples were in D_2O buffer (20 mM Tris 150 mM NaCl pD 8.0) at a concentration of ~0.8 mM and 40 µL was placed between two CaF_2 windows with a 50 µm spacer and into a Harrick cell for the measurement. The light adapted state was generated by continuously illuminating the sample using a 455 nm high power mounted LED (Prizmatix, Ltd.) placed in the sample compartment and focused onto the cell. The dark state spectrum was subtracted from the light state spectrum to generate the light minus dark (L-D) difference spectrum. All measurements were performed at room temperature.

Ultrafast Time-Resolved Infrared Spectroscopy

Ultrafast time-resolved IR (TRIR) spectra were measured with ~ 100 fs time resolution at the STFC Central Laser Facility using the ULTRA apparatus described elsewhere.[2,3] TRIR spectra were acquired at 20 °C from 1400 – 1800 cm^{-1} at a resolution of 3 cm^{-1} per pixel. Data were obtained using a 50 µm path length flow cell which was also rastered in the excitation beam to minimize photochemistry (photobleaching, photodegradation and photoconversion). The excitation beam of the 450 nm 100 fs 5 kHz pulses was focused to a spot size of ~ 100 µm and the pulse energy was kept below 400 nJ to avoid build-up of sample in light state. Transient difference spectra (pump on – pump off) were recorded using the IR probe at time delays between 1 ps and 2 ns. After the measurements were recorded the extent of photoconversion was shown to be negligible using absorbance spectroscopy. Spectra were calibrated relative to the IR transmission of a pure *cis* stilbene standard sample placed at the sample position.

Time Resolved Multiple Probe Spectroscopy (TRMPS)

TRMPS spectra were obtained at 20 °C from 100 fs to 1 ms at the STFC Central Laser Facility. The TRMPS method has been described elsewhere,[3] and previously used by us to analyze the photoactivation of $AppA_{BLUF}$, and other photoactive and photochromic proteins.[1,4-6] Light sensitive samples were analyzed using a rastered flow cell, and data were acquired using a 450 nm pump pulses operated at 0.6-0.8 µJ per pulse and a repetition rate of 1 kHz. The spectral resolution was 3 cm^{-1} and the temporal resolution was 200 fs. A typical measurement was acquired during 45 min of data collection. All samples were prepared at 0.6-0.8 mM concentration in D_2O buffer prepared with 20 mM Tris, 150 mM NaCl, pD 8.0. TRIR and TRMPS data were globally analyzed using the sequential model in Glotaran.

Ultrafast Transient Absorption experiments

Ultrafast transient absorption measurements were performed using a Spitfire Ace regenerative amplifier system providing ~800 μJ pulses centered at 800 nm at a repetition rate of 1 kHz. The output of the amplifier was split in the ratio 1:9. The pulse with the smaller energy was used for generation of the white light continuum probe in a CaF_2 crystal. The higher intensity fraction was frequency doubled to 400 nm and attenuated to ~200-400 nJ/pulse before being used as the pump pulse. Polarization of the probe was again set to magic angle compared to excitation. To avoid photodegradation the samples were moved with the help of a Lissajous scanner and simultaneously flowed by a peristaltic pump. Absorption changes were measured with an Andor CCD and collected with the help of home written Labview data acquisition software, and are again reported as pump on – pump off normalized difference spectra.[7]

Adenylyl Cyclase Assay

The adenylyl cyclase (AC) assay was performed using continuous and discontinuous formats which both monitored the consumption of NADH at 340 nm. In each case the assays were performed on freshly purified protein.

Discontinuous Assay: AC activity was determined at 22 °C in a total reaction volume of 1.5 mL using 20 mM Tris, 150 mM NaCl, 1mM $MgCl_2$ pH 8.0 buffer and by varying the concentration of ATP from 10 μM to 700 μM. The reaction was initiated by adding OaPAC to give a final concentration of 1 μM and 120 μL samples were withdrawn at different time intervals (0, 10, 20, 30, 40, 50, 60, 90, 120, and 180 s) after which they were quenched by heating to 100 °C for 5 min. The reaction mixture was either kept in the dark, for the dark-state control, or continuously illuminated with a blue LED (peak wavelength at 455 nm and excitation intensity of 500 μW per cm^{-2}) after obtaining the 0 time point sample. After quenching, the samples were then filtered using a 0.22 μm filter (Millipore Sigma, PVDF, 33 mm) and the amount of PPi generated in the reaction was quantified using a pyrophosphate reagent kit from Sigma (P7275), in which the oxidation of NADH to NAD^+ was monitored at 340 nm. This was achieved by combining 66.7 μL of the filtered samples with 33.3 μL of the pyrophosphate reagent in a clear 96 well plate. The plate was incubated for 1 min at 37 °C and then the absorbance at 340 nm was recorded using a BioTek Synergy Neo2 multi-mode plate reader. The absorbance was monitored until no further change was observed, indicating complete conversion of the PPi liberated in the OaPAC reaction to NAD^+. The final A_{340} for each sample was plotted as a function of time to enable initial velocities to be extracted

by fitting the data to a straight line using OriginPro software. The experiment was repeated at multiple ATP concentrations (10 to 600 μM) and V_{max} and K_m parameters were determined by fitting the initial velocity data obtained as a function of ATP concentration to the Michaelis-Menten equation using GraphPad Prism 9 software. A similar protocol was followed for each of the OaPAC n-FY6 variants. For each protein sample, the experiment was performed in duplicates and the average of data plotted with the standard error of the mean (SEM).

Continuous assay: Samples were prepared as described above except that the production of PPi was directly monitored by combining 250 μL of the pH 8.0 OaPAC/ATP reaction mixture with 125 μL of the reconstituted pyrophosphate reagent before illuminating the sample with light. The reaction mixture was incubated in a water bath at 30 °C for 1 min then 100 μL was placed in a sub-micro quartz cuvette. Using an Ocean Optics USB2000+ spectrometer, the absorbance at 340 nm was recorded for 40 s before the sample was illuminated using the blue LED (see above). The reaction was continuously monitored for at least 4 min and initial velocities were extracted for each ATP concentration from a plot of A_{340} vs time. Values for V_{max} and K_m were calculated by fitting the initial velocity data obtained as a function of ATP concentration to the Michaelis-Menten equation using GraphPad Prism 9. The same steps were performed for the n-FY6 variants, however for the 2,3-F_2Y6, 3,5-F_2Y6 and 2,3,5-F_3Y6 variants a background rate in the presence of blue light and in the absence of ATP was observed, which was subtracted from the initial velocities obtained in the presence of ATP. The data reported for the continuous assay are the averaged mean of 2 replicates with their standard error of the mean (SEM).

References

(1) Gil, A. A.; Haigney, A.; Laptenok, S. P.; Brust, R.; Lukacs, A.; Iuliano, J. N.; Jeng, J.; Melief, E. H.; Zhao, R. K.; Yoon, E.; Clark, I. P.; Towrie, M.; Greetham, G. M.; Ng, A.; Truglio, J. J.; French, J. B.; Meech, S. R.; Tonge, P. J. (2016) Mechanism of the AppABLUF Photocycle Probed by Site-Specific Incorporation of Fluorotyrosine Residues: Effect of the Y21 pKa on the Forward and Reverse Ground-State Reactions *J Am Chem Soc*, **138**, 926-935. DOI: 10.1021/jacs.5b11115

(2) Greetham, G. M.; Burgos, P.; Cao, Q.; Clark, I. P.; Codd, P. S.; Farrow, R. C.; George, M. W.; Kogimtzis, M.; Matousek, P.; Parker, A. W.; Pollard, M. R.; Robinson, D. A.; Xin, Z. J.; Towrie, M. (2010) ULTRA: A Unique Instrument for Time-Resolved Spectroscopy *Appl Spectrosc*, **64**, 1311-1319. DOI: 10.1366/000370210793561673

(3) Greetham, G. M.; Sole, D.; Clark, I. P.; Parker, A. W.; Pollard, M. R.; Towrie, M. (2012) Time-resolved multiple probe spectroscopy *Rev Sci Instrum*, **83**, 103107. DOI: 10.1063/1.4758999

(4) Brust, R.; Lukacs, A.; Haigney, A.; Addison, K.; Gil, A.; Towrie, M.; Clark, I. P.; Greetham, G. M.; Tonge, P. J.; Meech, S. R. (2013) Proteins in action: femtosecond to millisecond structural dynamics of a photoactive flavoprotein *J Am Chem Soc*, **135**, 16168-16174. DOI: 10.1021/ja407265p

(5) Laptenok, S. P.; Gil, A. A.; Hall, C. R.; Lukacs, A.; Iuliano, J. N.; Jones, G. A.; Greetham, G. M.; Donaldson, P.; Miyawaki, A.; Tonge, P. J.; Meech, S. R. (2018) Infrared spectroscopy reveals multi-step multi-timescale photoactivation in the photoconvertible protein archetype dronpa *Nat Chem*, **10**, 845-852. DOI: 10.1038/s41557-018-0073-0

(6) Iuliano, J. N.; Collado, J. T.; Gil, A. A.; Ravindran, P. T.; Lukacs, A.; Shin, S.; Woroniecka, H. A.; Adamczyk, K.; Aramini, J. M.; Edupuganti, U. R.; Hall, C. R.; Greetham, G. M.; Sazanovich, I. V.; Clark, I. P.; Daryaee, T.; Toettcher, J. E.; French, J. B.; Gardner, K. H.; Simmerling, C. L.; Meech, S. R.; Tonge, P. J. (2020) Unraveling the Mechanism of a LOV Domain Optogenetic Sensor: A Glutamine Lever Induces Unfolding of the Jalpha Helix *ACS Chem Biol*, **15**, 2752-2765. DOI: 10.1021/acschembio.0c00543

(7) Snellenburg, J. J.; Laptenok, S. P.; Seger, R.; Mullen, K. M.; van Stokkum, I. H. M. (2012) Glotaran: A Java-Based Graphical User Interface for the R Package TIMP *J Stat Softw*, **49**, 1-22. DOI: 10.18637/jss.v049.i03
